# A hybrid gazelle optimization and reptile search algorithm for optimal clustering in wireless sensor networks

**DOI:** 10.1038/s41598-025-96966-9

**Published:** 2025-04-26

**Authors:** Soha S. Elashry, A. S. Abohamama, Hatem Mohamed Abdul-Kader, M. Z. Rashad, Ahmed F. Ali

**Affiliations:** 1 Department of Computer Science, Faculty of Computers and Information, Mansoura University, Mansoura, Egypt; 2 Department of Information System, Faculty of Computers and Information, Menofia University, Shebien El Koum, Egypt; 3 Faculty of Information Technology and Computer Science, Sinai University, Kantara, Egypt; 4 Department of Computer Science, Arab East Colleges, Riyadh, Saudi Arabia; 5 Department of Computer Science, Faculty of Computers and Informatics, Suez Canal University, Ismailia, Egypt

**Keywords:** Clustering, Meta-heuristics, Wireless sensor network (WSN), Network lifetime, Energy consumption, Reptile Search Algorithm (RSA), Gazelle Optimization Algorithm (GOA), Computer science, Information technology, Software

## Abstract

In our modern societies, the wireless sensor network (WSN) is categorized as a smart motivated technology that can be utilized in many work environments and activities to enhance daily life. However, several challenging concerns have been assigned to WSN. The clustering process is a main complex concern and still an open problem in WSN. To support an efficient clustering process, two crucial requirements must be considered, energy management and network lifetime extension, especially in the development of large-scale WSN. The primary objective of this article is to introduce a new meta-heuristic algorithm, denoted as the hybrid gazelle optimization and reptile search algorithm (HGORSA), which optimizes cluster head selection in WSNs. In the proposed algorithm, the mathematical models for the exploration and exploitation phases of the traditional gazelle optimization algorithm (GOA) are enhanced by integrating the hunting operator, reduction function, and predator cumulative effect operators from the traditional RSA. These modifications improve the balance between diversification and intensification processes, effectively addressing two key clustering requirements mentioned above. At the same time, they also positively impact the overall performance evaluation of the WSN. Various simulation scenarios are designed to evaluate the performance of the proposed HGORSA in different network configurations. First, the main experiment was conducted with 300 sensor nodes (SNs). The experimental results then analyzed to assess the effectiveness of the proposed algorithm under different conditions against six state-of-the-art meta-heuristic algorithms. Based on simulation outputs, HGORSA demonstrated superior performance compared to particle swarm optimization, grey Wolf optimizer, sperm swarm optimization, chernobyl disaster optimizer, gazelle optimization algorithm and reptile search algorithm. Specifically, HGORSA achieved percentage improvements in terms of stability period (37.3%, 49.6%, 46.8%, 55.3%, 19.1%, and 34.4%, respectively), energy consumption (10.8%, 10.5%, 9.6%, 8.6%, 8.3%, and 3.5%, respectively), network lifetime (44.5%, 40.8%, 23.8%, 16.8%, 9.3%, and 7.2%, respectively), reduction in number of dead nodes (30.3%, 29.7%, 28.9%, 24.3%, 18%, and 11.5%, respectively), and network throughput (36.4%, 43.9%, 34.2%, 25%, 20%, 14.4%, respectively). Moreover, a supplementary experiment was conducted to test the efficiency of the HGORSA algorithm in dense and sparse networks, where the number of SNs was set at 50 and 500. The algorithm was evaluated based on the five standard aforementioned performance metrics. Furthermore, the robustness of HGORSA was validated using statistical measures, including standard deviation (Std), average (Avg), worst and best values, and box plots of the fitness function across 20 independent runs. Based on statistical results, HGORSA outperformed the other comparative meta-heuristics.

## Introduction

**Fig. 1:**
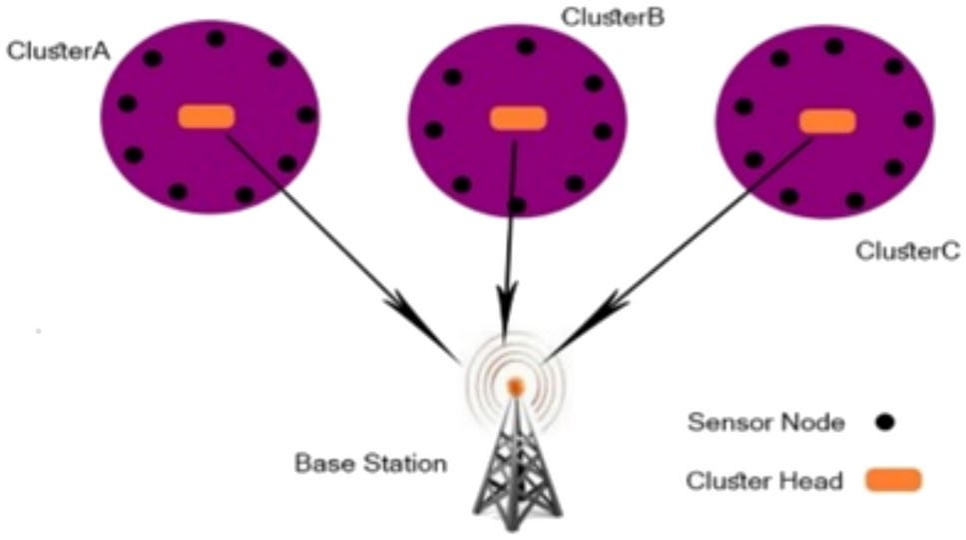
WSN based-clustering. The figure was prepared using Adobe Photoshop version CS6. https://www.adobe.com/eg_en/products/photoshop/online.html.

The world of telecommunications and intelligent engineering is currently experiencing a remarkable transformation, driven by several factors, among them the extensive use and achievements of wireless sensor technology. These wireless sensors basically are used to collect sensed data from their surrounding environment and have the benefit of being small in magnitude and easy to assemble, even in challenging conditions. Furthermore, they are fundamental elements in the WSN, 5G, and Internet of Things (IoT) domain^1^. However, optimal wireless sensors management (optimal clustering) in WSN is a main concern as it generally makes WSN performs better especially, considering energy consumption and network lifetime extension. From an industrial, commercial, and social point of view, WSN is considered intelligent technology driven by the aim of improving people’s lives. Due to its numerous uses in a range of industries, including traffic monitoring^2,3^, agriculture^4^, Internet of Drones^5^, Internet of Food^6^, Internet of Medical Things^7^, vehicle automation^8^, and Industrial IoT (IIoT)^9^. Furthermore, WSN and IoT technology can be effectively combined as a unified system, allowing widespread utilization^10^. From a research community point of view, although WSN is extensively investigated in many research works, it remains a leading topic of discussion in a wide variety of works In WSN, the long journey of data collected dissemination from the source (SNs) to the destination (detonated as the sink ) must be efficiently managed. One of the most important techniques for managing this journey with a high rate of optimizing the performance of WSN is the clustering mechanism. For WSN-based clustering, this journey begins with smart objects termed SNs, which are distributed within the same communication range and are connected to supervisor nodes termed CHs, or cluster heads. CHs are in charge of data aggregation, removing correlated and unwanted data, and finally sending data to the destination, which is a remote location denoted sink^11–13^. This technique is represented in Fig. 1. The clustering challenge in WSNs is an NP-hard problem (nondeterministic optimization problem ) in nature. Using meta-heuristic algorithms is the best solution to handle these categories of complex optimization^14^. In addition, meta-heuristic algorithms can be used to solve real-world problems, such as the traveling salesman problem (TSP)^15^, cardiac arrhythmia, and other important biomedical applications^16^. They are also applied in various engineering fields^17,18^, and in solving multi-objective optimization problems^19,20^, and^21^. Plethora of meta-heuristic algorithms utilized in clustering concern in the WSN. This article is based on GOA^22^ and RSA^23^ which are examples of efficient and recent meta-heuristic algorithms. To improve the performance of meta-heuristic algorithms, in this article the hybridization algorithm approach^24^ is utilized. The proposed algorithm derived from them is a better choice for such these challenging problem categories due to the ability to escape from the local optimum and quick convergence and high quality of solution for the clustering optimization problem in WSN while maintaining the desirable performance of the network.

However, Literature reveals that one of the main challenges of existing clustering protocols based on meta-heuristic algorithms is their high energy consumption, which can lead to a shortage network lifetime. This article mainly introduces a new hybrid meta-heuristic algorithm of optimal clustering in WSN and maintaining low energy consumption and lengthening the lifetime of the network. The proposed algorithm integrates between gazelle optimizer and reptile search algorithm, denoted as the hybrid gazelle optimization and reptile search algorithm (HGORSA). Due to the efficiency of the two algorithms, they have been applied to solve many problems^25–29^. The integration of the two algorithms improves both the exploration and exploitation phases of the traditional GOA. Additionally, the proposed algorithm is competent since it achieves the optimal global solution while avoiding being stuck in local optima to be highly effective in clustering optimization problem in WSN. Numerical experiments have shown that HGORSA enables significant network performance by achieving energy conservation, maximizing WSN lifetime, a decrease in number of dead nodes, more network throughput, and an extended stability period compared to the performance of the other comparative meta-heuristic algorithms.

The proposed HGORSA has practical implications across various fields. In the environment, it can be used to monitor conditions such as temperature, humidity, air quality, soil moisture, and water levels. In healthcare, it facilitates remote health monitoring and enables real-time data analysis for faster diagnosis and treatment, particularly in remote or undeserved areas. In agriculture, it helps to monitor soil conditions, weather patterns, crop health, and more. Despite all of the advances in the problem-solving abilities of the proposed algorithm, Wolpert and Macready’s no-free-lunch theorems^30^ demonstrate that it is unrealistic to anticipate a general problem solver that can solve any optimization problem.

The key contributions of this article are as follows.

- To our knowledge, this is the first work in which the GOA is implemented to optimize the CH clustering problem in WSNs .
- Introducing a new hybrid meta-heuristic algorithm, denoted as HGORSA, which integrates the phases of GOA and RSA for an efficient clustering process in WSN.
- Interpreting statistical tests in terms of standard deviation (Std), average (Avg), worst, and best values to assess the robustness of HGORSA by comparing it with six state-of-the-art meta-heuristic algorithms.
- Making a valuable contribution to the field, particularly in five different standard performance metrics, including network lifetime, energy consumption, number of dead nodes, network throughput, and network stability period.
- Evaluating the efficiency of the HGORSA algorithm on both dense and sparse networks using the five mentioned performance metrics. Specifically, the algorithm is tested with 50 and 500 sensor nodes as a supplementary experiment, along with the main experiment with 300 sensor nodes.
- Providing box plots of the fitness function across 20 independent runs, which considers an important measurement of the performance of the meta-heuristic algorithms. The structure of the rest of this article is the following organization. Section "Literature review" briefly presents the background of the article for some of the meta-heuristic algorithms that are exploited in WSNs. The problem definition presented in Section "Problem definition", and the GOA and RSA algorithms are covered in Section "The Gazelle optimization and reptile search algorithms". The details of the proposed HGORSA are explained in Section 5. The simulation outputs are investigated and evaluated in Section "Numerical results and discussion". The conclusion of this article and possible future visions are presented in Section "Conclusion and future work".

## Literature review

In general, meta-heuristic algorithms are presented as perfect and low-cost optimal solutions to solve complex problems. Among these problems, designing cluster-based algorithms for WSN has a strong influence on energy saving, network lifetime extension, and generally high network performance. This section will investigate WSN clustering and routing, concentrating on employing meta-heuristic algorithms to solve this challenge. In this section, the reviewed papers are compared to clarify the research gap.

In 2020, in^31^, a CH-based energy-aware optimized routing approach based on the sailfish optimizer (SFO) and multi-objective function was introduced for the WSNs. MCH-EOR effectively optimized power consumption and improved the overall performance of the network than other existing models.

In 2021, a meta-heuristic clustering algorithm is proposed based on particle swarm optimization (PSO) and genetic algorithm (GA) for WSns in^32^. In this algorithm, GA is used to choose the appropriate CHs, and PSO to identify the best possible route for the CHs to the sink. Outperformed state-of-the-art algorithms in five performance metrics.

In 2022, a novel framework for the selection of CHs in WSNs is proposed that considers energy consumption through hierarchical routing, employing sea lion optimization (SLnO) and PSO in^33^. The selection process has considered few elements, including distance, delay, quality of service (QoS), and energy.

Another hybrid approach in^34^ integrated the differential evolution algorithm (DE) and the sparrow search algorithm (SSA) to address the energy efficiency problem related to the selection of CHs in WSN. The performance of the proposed method is evaluated over that of other traditional methods in four standard metrics.

In addition, researchers in^35^ have suggested an approach called MPO-IPSO-OCR, which combines the pros of PSO and marine predator optimization (MPOA) to achieve efficient CH election and data transmission. This method is compared with the other schemes in terms of throughput, network lifetime, and energy consumption.

The work in^36^ combined the fruitfly optimization algorithm (FFOA) and the glowworm swarm optimization (GSO) to identify the most suitable CH in WSN. The evaluation of this algorithm is done with a comparison against many different existing algorithms in terms of active nodes, energy utilization, and a cost function.

In 2023, in^37^, a distributed protocol named DPFCP (distributed particle swarm optimization-based fuzzy clustering protocol) was developed for WSNs. The simulation results have shown a significant improvement in the longevity and power consumption of the network compared to the available techniques.

In 2024, in^38^, a hybrid algorithm called MMMRA (multi-objective optimized multi-path and multi-hop routing algorithm) is proposed for efficient routing in WSNs. It combined the chimp optimization algorithm (COA) with the ant colony optimization (ACO). Outperformed competitors in residual energy, HND and FND metrics.

The authors in^39^ modified the artificial fish swarm algorithm (MAFS) to optimally group nodes with the weighted k-means clustering algorithm. In this work, a great magnitude of efforts have been reported to acquire energy efficiency in WSN; these efforts range from conventional approaches to meta-heuristic approaches to enhance network performance.

In addition, in 2024, Elashry et al.^40^ proposed a novel nature-inspired technique that denoted the chaotic reptile search algorithm (CRSA). CRSA is a combination of chaotic map and reptile search optimizer. Simulation has shown that CRSA has achieved better performance than eight methods according to four standard evaluation metrics.

The authors in^41^ proposed a greywolf optimization algorithm that helps to achieve energy efficiency in the network. The results showed that the GWO-P beats the contender calculations for the length of stability and network lifetime.

In order to extend the lifespan of a heterogeneous WSN, the author in^42^ suggested an algorithm that makes use of adaptive crossover and binary tournament selection techniques. The simulation results showed that the suggested algorithm significantly improves network stability and operating time compared to other current methods.

The author in^43^ suggested a technique throughout combining the PSO with the GA, to calculate the probability of selecting the best nodes as CH in the WSNs. This method performed better in energy consumption, network overhead, packet delivery ratio, throughput, and end-to-end delay than other existing methods.

For heterogeneous IoT networks, the author in^44^ introduced a novel clustering protocol based on GA for WSN. The simulation findings revealed that the proposed method achieved improvements over other approaches in terms of network lifetime, number of alive nodes, residual energy, and network stability.

In^45^, the author presented an approach to determine the likelihood of selecting the best nodes to lead the cluster, and the bat optimization algorithm (BAT) is used to find the shortest path. This method achieved improvements in terms of network stability of throughput, network longevity, and throughput over traditional methods.

The research gaps highlighted in Table 1 clarify the need for an efficient algorithm that ensures high network stability and high overall performance for the network, scaling with increasing network size, adapting to the three sink locations (corner, center and outside field deployment area ) in the simulation process, and showing high efficiency in both statistical and simulation findings.

The motivation for this article stems from the fact that WSN remains a prominent topic of discussion in many publications, despite being extensively studied in previous researches. Effective management of data transmission over long distances, from the SNs to the destination (the sink), is a critical requirement for WSNs. The clustering mechanism is one of the most important methods for optimizing this process, significantly enhancing the performance of WSN.

The related studies can be summarized in Table 1 as follows.

**Table 1:** Summary of related studies.

Year	The applied algorithm	Pros	Cons
2020	SFO.	This method optimized power consumption and improved the overall performance of the network.	Different sink location is not taken into account.
2021	PSO and GA.	This method outperformed the state-of-the-art algorithms in five performance metrics.	High computational complexity.
2021	AFS.	It enhanced the energy efficiency in WSN than other traditional approaches.	Difficult to predict k-value.
2021	GWO.	The performance metrics of this method compared over the existing algorithms in terms of network stability and network lifetime.	The efficiency does not test in large-scale WSN.
2022	PSO and SLnO.	This method the other models in terms of energy consumption and number of alive nodes.	The performance is not efficient.
2022	SSA.	The performance of the proposed method is evaluated over that of other traditional methods in four standard metrics.	The statistical analysis is not taken into account.
2022	PSO and MPOA.	This method is compared with the other schemes in terms of throughput, network lifetime, and energy consumption.	The scenario is be implemented on smaller network.
2022	FFOA and GSO.	The evaluation of this algorithm is done with a comparison against many different existing algorithms in terms of active nodes, energy utilization, and a cost function.	The sink is only positioned at one location.
2022	GA.	The simulation results show that the suggested algorithm significantly improves network stability and operating time compared to current methods.	The sink mobility in the network need to taken into account.
2023	PSO.	The simulation results have shown a significant improvement in the longevity and power consumption of the network compared to the available techniques.	CHs communicate with the sink in single-hop mode.
2023	GA.	The proposed method achieved improvements over other approaches in terms of network lifetime, number of alive nodes, residual energy, and network stability.	The statistical analysis not included.
2024	COA and ACO.	This method outperformed the competitors in residual energy, HND and FND metrics.	Scenario of the heterogeneous network needed to be considered.
2024	CRSA.	Simulation has exhibited that CRSA has gained better performance than eight methods according to four standard evaluation metrics.	Network stability need to be enhanced.
2024	PSO and GA.	This method performed better in energy consumption, network overhead, packet delivery ratio, throughput, and end-to-end delay than other existing methods.	The simulation results are compared with only three existing methods.
2024	BAT.	This method achieved improvements in terms of network stability of throughput, network longevity, and throughput over traditional methods.	The efficient utilization of the energy harvesting enabled nodes needs to be considered.

## Problem definition

Designing a cluster-based algorithm which involves determining the best option for CHs and the formation of clusters is a challenging task for WSNs. This clustering process affects efficient energy consumption control, better network lifetime, and generally high network performance. Our algorithm clusters data in two stages: initially, CHs are selected, and secondly, clusters are formed. In the following subsections, an orientation for these two steps will be discussed.

To better understand the proposed cluster head selection process, the terms used will be clarified first as shown in Table 2.

**Table 2:** The notations in the CHs selection phase.

Notation	Purpose
*CH*	Cluster head.
*C*	Set of the cluster heads.
*m*	Count of CHs.
$s_i$	Set of SNs.
*N*	Count of SNs.
*BS*	Base station or the sink.
$R_{max}$	CH maximum communication range.
*R*	SN maximum communication range.
$TH_{E}$	Threshold energy for being a CH.
$E_{CH_j}$	Energy value for a cluster head *j*.
$\alpha$	Control factor.

### The select of CHs

In the proposed algorithm, the CHs are selected by applying a distinct fitness function that is determined by several parameters, as follows:

- **Mean range among SNs and CHs.** It is the total distances between all SNs $s_i$ and every CH $j(CH_j)$. Next, their mean is computed as indicated by Eq. (1).
  1 $$\begin{aligned} \frac{1}{m}\sum _{i=1}^{N} distance(s_i,CH_j) \end{aligned}$$
  Whereas *N* represents the count of sensor nodes and *m* indicates the count of CHs.
- **Mean range between CHs and sink/ base station (BS).** Eq. (2) states that it indicates the distance, divided by the count of CHs (*m*), between each CH $j(CH_j)$ and the BS (*BS*).
  2 $$\begin{aligned} \frac{1}{m}\; distance(CH_j,BS) \end{aligned}$$
  Every CH aggregates information sensed directly from its SNs and begins to send it to the base station. Thus, choosing the CHs that are closest to the BS is preferable. To reduce the separation between nodes and cluster heads and the distance between the base station and each cluster head, the Eqs. (1), (2) in (3) (dubbed $f_{distance}$) have been merged.
  3 $$\begin{aligned} \begin{aligned} Min\;\; f_{distance}= \sum _{j=1}^{m} \frac{1}{m}&\bigg (\sum _{i=1}^{N} distance(s_i,CH_j)+ distance(CH_j,BS) \bigg ) \end{aligned} \end{aligned}$$
  item **CHs’ total energy** The total energy of the current for every chosen CH is indicated by this value. Our goal is to select the best CHs by maximizing this total. In other words, the inverse of this sum needed to be minimized, which is represented by $f_{energy}$ in Eq. (4). Since each node uses energy during data transmission, select of the CHs with more energy ownership than other nodes is vital.
  4 $$\begin{aligned} Min\;\; f_{energy}= \frac{1}{\sum _{j=1}^m(E_{CH_j})} \end{aligned}$$
  Energy value for a cluster head *j* is $E_(CH_j)$, where $(1\le j \le m)$.

Equation (5) illustrates how to combine the previous two functions, ${f_{distance}}$ and ${f_{energy}}$, to generate the fitness function, which is designated ${F_{fitness}}$.

5 $$\begin{aligned} Min \quad F_{fitness}=&\alpha \times f_{distance} + (1-\alpha ) \times f_{energy} \nonumber \\ \text {s.t.} \quad&\nonumber \\&distance(s_i,CH_j )\le R \;\; \forall s_i \in SNs, \;\; CH_j \in C \nonumber \\&distance(CH_j,BS)\le R_{max}\;\;\forall CH_j \in C \nonumber \\&E_{CH_j}> TH_E, \;\;\; 1\le j \le m \nonumber \\&0< \alpha<1\nonumber \\&0<f_{distance},f_{energy}<1 \end{aligned}$$

For each SN, *R* represents its maximum communication range. For each CH, the values $s_i$, $R_{max}$, *N*, *C*, and $C =\{CH_1,CH_2,\ldots ,CH_m\}$ indicate the greatest communication range. The threshold energy for a CH is denoted by $TH_E$, and the control parameter is $\alpha$.

The main goal is to minimize the value of the fitness function in Eq. (5) to select the optimal CHs. The best CH situation is when the level of fitness is reduced.

### Creation of clusters

After the completion of the first stage, the clusters begin to form. The cluster creation process employs a weight function known as *WeightF*, which depends on subsequent parameters:

- **The CH’s residual energy.** A CH $j(CH_j)$ with the greater remaining energy compared to other CHs within its range of communication should result from the combination of an SN $s_i$. As a result,
  6 $$\begin{aligned} WeightF(s_i,CH_j) \propto E_{residual}(CH_j) \end{aligned}$$
  The residual energy for a CH *j* is indicated by $E_{residual}(CH_j)$.
- **The CH’s distance from the SN.** If the SN $s_i$ is within the communication range of a CH $j(CH_j)$, it should combine with it. Doing so will help reduce the use of energy. As a result,
  7 $$\begin{aligned} WeightF(s_i,CH_j) \propto \frac{1}{distance(s_i,CH_j)} \end{aligned}$$
- **The CH’s distance from the BS.** The data must be received by the CHs from the SNs and forwarded to the BS. Because of this, an SN $s_i$ has to unite to form a cluster head that is closer to the base station than other cluster heads within reach.
  8 $$\begin{aligned} WeightF(s_i,CH_j) \propto \frac{1}{distance(CH_j,BS)} \end{aligned}$$
- **The CH node’s degree.** It ought to come together to a CH $j(CH_j)$ that, among the nodes within its communication range, has the lowest degree for an SN $s_i$. Due to this,
  9 $$\begin{aligned} WeightF(s_i,CH_j) \propto \frac{1}{node\_{degree}(CH_j)} \end{aligned}$$
  Equation (10) may be created by combining Eqs. (7), (8), and (9).
  10 $$\begin{aligned} \begin{aligned} WeightF(s_i,CH_j)\propto \frac{E_{residual(CH_j)}}{distance(s_i,CH_j)} \times \frac{1}{distance(CH_j,BS)} \times \frac{1}{node\_{degree}(CH_j)} \end{aligned} \end{aligned}$$
  As a result, the cluster formation final weight function is found in Eq. (11)
  11 $$\begin{aligned} \begin{aligned} WeightF(s_i,CH_j)= C \times \frac{E_{residual(CH_j)}}{distance(s_i,CH_j)} \times \frac{1}{distance(CH_j,BS)} \times \frac{1}{node\_{degree}(CH_j)} \end{aligned} \end{aligned}$$
  where a constant denoted by *C* has a value of 1. Every SN decides its *WeightF* using Eq. (11), and then combines to form a CH with the highest weighted value to build the clusters.

## The Gazelle optimization and reptile search algorithms

### Background for gazelle optimization algorithm (GOA)

The gazelle optimization algorithm (GOA) is a nature-inspired algorithm developed by Agushaka et al.^22^. GOA simulates the natural behavior of gazelle life and how they graze and avoid being hunted by predators such as hyenas, Asiatic, lions, and leopards. By following subsections, the GOA’s main phases and how it operates are represented.

### Inspiration and the natural behaviors

Gazelles reside in drylands that cover most of Asia, comprising parts of the Arabian Peninsula, China, and the northern Sahara Desert in Africa. In addition, gazelles are widespread in the sub-Saharan Sahel, which extends from Tanzania to the African Horn and northeastern Africa. In general, gazelles are hunted by predators regularly, and they are fast-moving animals with acute senses of smell, sight, and hearing. To compensate for their apparent recurring vulnerabilities, these adaptive features allow them to escape from their predators. The unique characteristics of the gazelles’ behaviors can be seen. As herbivorous creatures, gazelles only consume plant materials like grasses, leaves, shoots, and other plant-based foods. They become accustomed to living in groups for safety and social reasons, like the majority of consumers. There can be as many as 700 members in one group, and they interact among themselves. A group of gazelles with many members can achieve herd security. With Thomson’s gazelles, it is typical for the social organization of the gazelle herd to be based on gender, with the females living in a smaller group with their fawns. The sole males in the bachelor’s herd, on the other hand, aid in protecting and providing for the group. Humans, cheetahs, Asiatic and black-backed jackals, wild dogs, spotted hyenas, lions, and leopards are common predators of gazelles^46^. Gazelles warn each other of an impending danger by flicking their tails, stamping their feet, or leaping into the air. Stotting occurs when a person is agitated or excited, though it is exactly unclear what causes it. The gazelle can run at a top speed of 100 km/hr, which is uncommon for lions and cheetahs, among other predators. Even though they are the fastest land predators, cheetahs cannot outrun or outmaneuver gazelles. Without an element of surprise, the gazelle would typically outrun and outmaneuver the predator at a rapid rate of speed, making the success of most predators dependent on how well they stalk the gazelle quietly. The strategies utilized by gazelles to survive are described in the following points, which would be used to simulate the suggested algorithm.

- The two most incredible activities are grazing and fleeing predators. In the absence of predators, the grazing feature can be exploited. Predators observe gazelles as they graze. The gazelle uses stotting to identify predators, among other things (2 m in height).
- Exploration can be used to outrun predators that have been spotted and reach a haven. Despite being slower than the quickest predator, it can outmaneuver it at 88 km/hr.

### The modeling of the GOA

The GOA is modeled after the survival strategy of gazelles. The optimization process includes grazing in the absence of a predator and fleeing to a haven when seen. The main phases of GOA are illustrated in the following subsections.

#### The initial population

Gazelles *X* with randomly initialized solutions are used in the population-based optimization technique, known as GOA. According to Eq. (12), the solutions are described as an $n \times d$ matrix of potential solutions.

12 $$\begin{aligned} X= \begin{bmatrix} x_{11}& x_{12}& \cdots & x_{1d} \\ x_{21}& x_{22}& \cdots & x_{2d} \\ \vdots & \vdots & x_{ij}& \vdots \\ x_{n1}& x_{n2}& \cdots & x_{nd} \end{bmatrix} \end{aligned}$$

Whereas *n* is the number of solutions in the population, the dimension of the problem is *d*, and $x_{ij}$ is a vector variable of a solution *i* in the dimension *j*.

#### The elite population

Each solution in the population is evaluated based on the objective function and the overall best solution is assigned and added to the elite matrix as follows.

13 $$\begin{aligned} E= \begin{bmatrix} e_{11}& e_{12}& \cdots & e_{1d} \\ e_{21}& e_{22}& \cdots & e_{2d} \\ \vdots & \vdots & e_{ij}& \vdots \\ e_{n1}& e_{n2}& \cdots & e_{nd} \end{bmatrix} \end{aligned}$$

As a result, the superior gazelle for building a leader matrix *E* as shown in Eq. (13) is selected as the solution that has been obtained most successfully so far. The gazelles use this matrix to look for and determine their next move.

#### The exploitation (intensification) phase

**Fig. 2:**
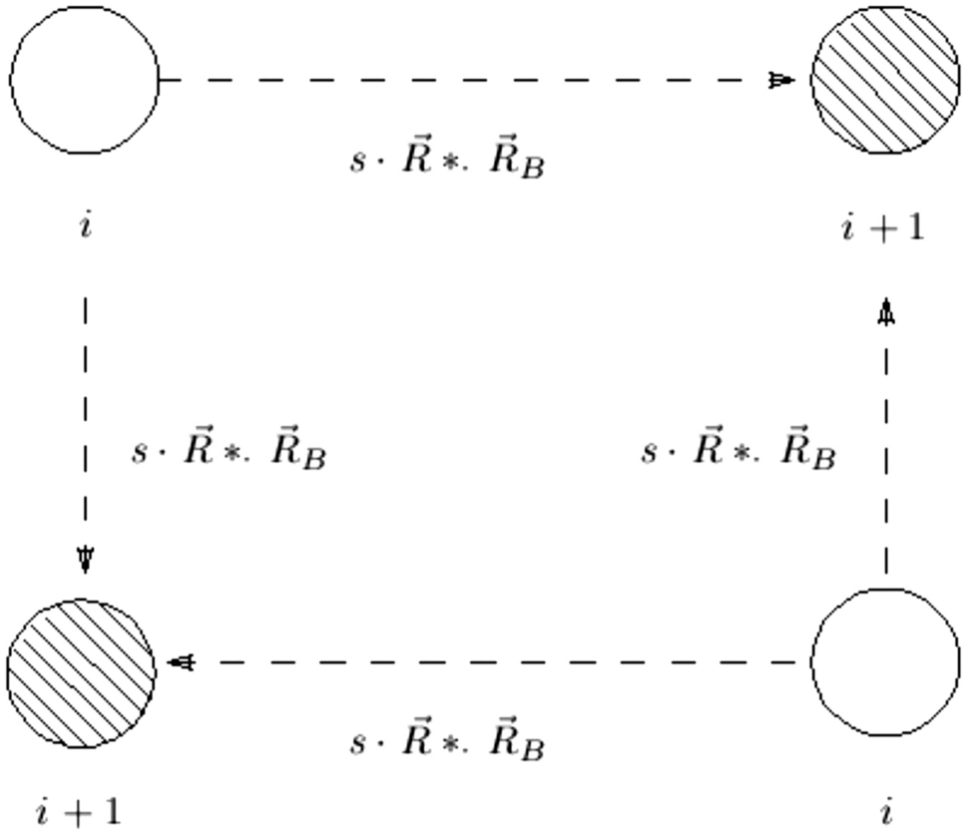
The gazelles’ grazing patterns are signs of the exploitation phase.

It is believed at this point that there is no predator or the predator is merely observing the gazelles as they peacefully graze. Using consistent and controlled steps of Brownian motion^47^, the neighborhood portions of the domain were efficiently covered during this phase. Gazelles are believed to move in Brownian motion while grazing, as seen in Fig. 2. The mathematical model illustrating this behavior is given by (14).

14 $$\begin{aligned} X_i^{t+1} = X_i^{t} + s\cdot R*\cdot R_B *\cdot (E_i- R_B*\cdot X_i^{t}) \end{aligned}$$

Where $X_i^{t+1}$ is a solution in the next iteration, $R_B$ consists of a vector of random numbers that represent the Brownian motion, $E_i$ is an elite solution in the elite matrix, *s* represents how quickly the gazelles are grazing., and the vector *R* contains uniform random numbers in [0,1].

#### The exploration (diversification) phase

**Fig. 3:**
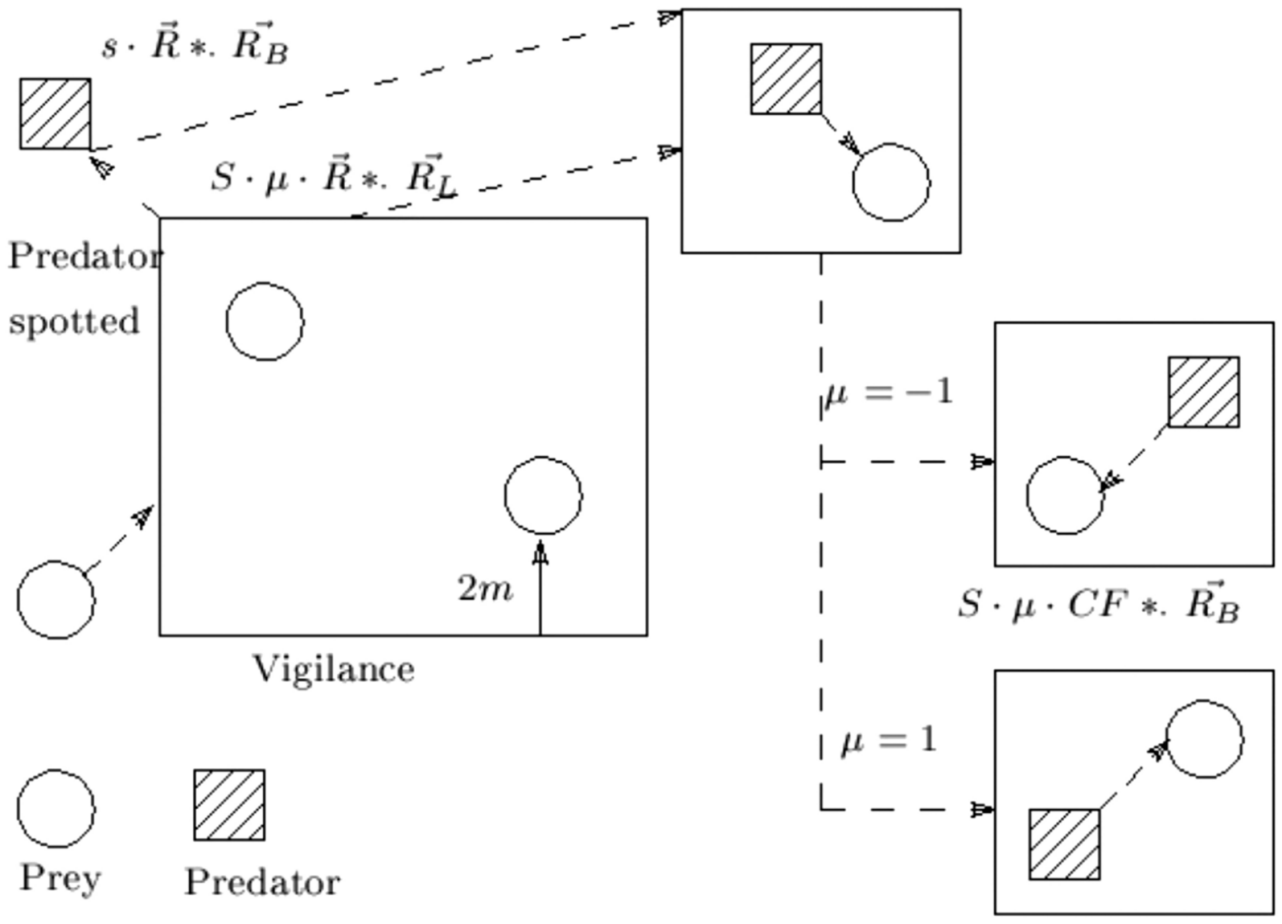
A gazelle runs from a predator (exploration phase).

This phase starts when a predator is spotted. Gazelles react to danger by flicking their tail, stomping their feet, or stotting up to 2*m* in the air with the four feet. The height 2*m* is simulated by scaling it to a number between 0 and 1. The Le’vy flight, which consists of a sequence of little steps and irregular big jumps, is used in this algorithmic phase. The search ability of the optimization literature has improved with this strategy^48^. In Fig. 3, the exploration phase is shown.

The gazelle runs away as soon as it spots the predator and the predator follows. A rapid shift in direction, symbolized by the $\mu$, characterizes both runs. The gazelle travels in one direction when the iteration number is odd and in the opposite direction when it is even. This paper assumes that the direction shift occurs per iteration. Since the gazelle reacts first, it used Le’vy flight to flee. The study presumptively used the Brownian motion for the predator’s initial takeoff run before switching to the Le’vy flight afterwards. Equation (15) illustrates the mathematical model of the behavior of the gazelle after spotting the predator.

15 $$\begin{aligned} X_i^{t+1} = X_i^{t} + MS\cdot \mu \cdot R *\cdot R_L *\cdot (E_i- R_L*\cdot X_i^{t}) \end{aligned}$$

Where *MS* is the maximum speed a gazelle can go, the Le’vy distribution-based random number vector is represented by $R_L$, $E_i$ is an elite solution in the elite matrix, and $\mu \in [-1,1]$. The Eq. (16) illustrates the mathematical model for the predator’s pursuit of the gazelle.

16 $$\begin{aligned} X_i^{t+1} = X_i^{t} + MS\cdot \mu \cdot CF *\cdot R_B *\cdot (E_i- R_L*\cdot X_i^{t}) \end{aligned}$$

Where $CF = (1-\frac{t}{m_{itr}})^{(2\frac{t}{m_{itr}})}$ is the cumulative effect of the predator, *t* is the current iteration, and $m_{itr}$ is the maximum number of iterations.

#### The predator success rates (PSRs)

In addition, the authors of a study on Mongolian gazelles stated that although the species is not endangered, its yearly survival rate is 0.66, meaning that there are only 0.34 successful predator encounters^49^. Predator success rates, or PSRs, affect the gazelle’s ability to escape; as a result, the algorithm stays out of a local minimum. Eq. (17) describes how the PSRs effect is modeled.

17 $$\begin{aligned} X_i^{t+1} = {\left\{ \begin{array}{ll} X_i^{t}+CF[LB + R*\cdot (UB-LB)]*\cdot U & \hbox { if}\ r \le PSRs \\ X_i^{t}+[PSRs(1-r)+r](X_{r_1}^{t}-X_{r_2}^{t}) & \text {otherwise} \end{array}\right. } \end{aligned}$$

where $X_{r_1}^{t}$, $X_{r_2}^{t}$ are randomly selected solutions, *r* is a random number generated in the range [0, 1] to create a binary vector *U* such that

18 $$\begin{aligned} U ={\left\{ \begin{array}{ll} 0& \hbox { if}\ r \le 0.34\\ 1 & \text {otherwise} \end{array}\right. } \end{aligned}$$

#### The Pseudo-code of the GOA

The algorithm’s exploitation phase simulates gazelles quietly grazing while the predator is either absent or stalking them. When a predator is detected, GOA enters the exploration phase. The gazelle must outrun and outmaneuver the predator during this phase to reach a safe sanctuary. To identify the best answers to optimization problems, the two steps are repeated subject to termination conditions. The pseudo-code for the GOA shown below depicts the implementation flow of these stages determined by their respective mathematical models as reported in Algorithm 1.

**Algorithm 1:**
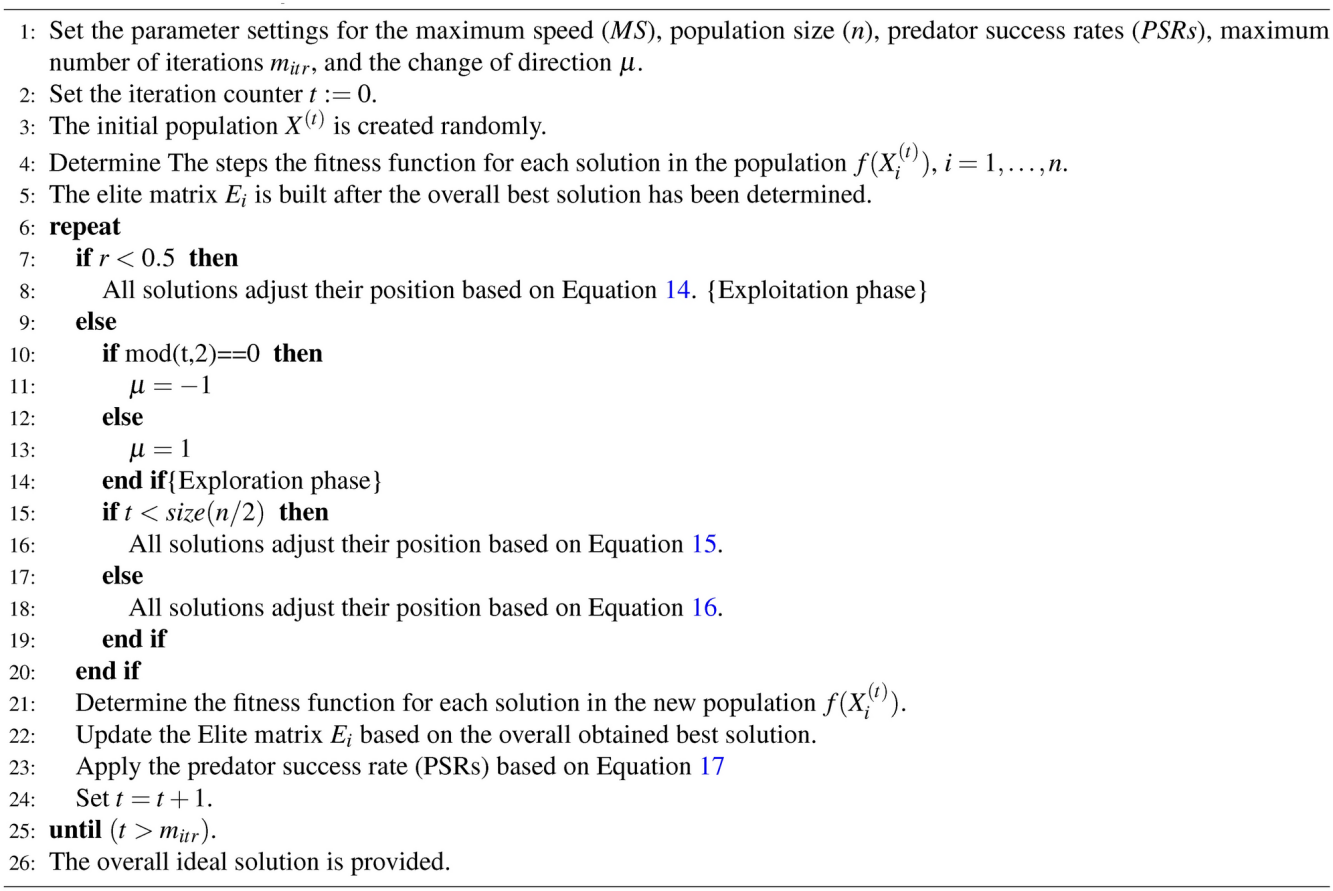
The GOA algorithm.

### Background on reptile search algorithm (RSA)

A population-based system called the Reptile Search Algorithm (RSA) simulates crocodile behavior throughout their regular hunting season using data from the natural world. Abualigah et al.^23^ introduced the RSA algorithm in 2022. The following sections explain the pseudo-code and key RSA steps.

#### The stage of initialization.

In RSA, the initial population begins with a set of candidate solutions (X) as indicated in Eq. (19), which is created stochastically and the best-obtained solution is considered to be roughly the optimum in each iteration.

19 $$\begin{aligned} X= \begin{bmatrix} x_{11}& x_{12}& \cdots & x_{1d} \\ x_{21}& x_{22}& \cdots & x_{2d} \\ \vdots & \vdots & x_{ij}& \vdots \\ x_{n1}& x_{n2}& \cdots & x_{nd} \end{bmatrix} \end{aligned}$$

Where *n* is the number of solutions in the population, *d* is the problem dimension, and $x_{ij}$ is a vector variable of a solution *i* in dimension *j*.

#### The exploration phase (encircling process).

According to their circling tendency, crocodiles move in two different ways: high walking and belly walking. These movements allude to many reigns with a dedication to exploration. Contrary to another search phase (the hunting phase), crocodile movements (such as high- and belly- walking) do not allow them to quickly approach the desired prey. The RSA can switch between the encircling (exploration) and hunting (exploitation) search phases. This behavior change is based on four conditions that are divided into four groups based on the total number of iterations. The two primary search techniques (high walking strategy and belly walking strategy) are used by the RSA exploration process to explore the search regions and approach to discover a better solution. There are two phases in which to move on to this stage of the search. The belly walking movement phase is conditioned by $t \le 2 \frac{m_{itr}}{4}$ and $t > \frac{m_{itr}}{4}$, while the high walking movement phase is conditioned by $t \le \frac{m_{itr}}{4}$ as shown in Eq. (20).

20 $$\begin{aligned} x_{ij}^{t+1} = {\left\{ \begin{array}{ll} x_{j}^{*t} \times -\chi _{ij}^{t} \times \nu - \gamma _{ij}^{t} \times r & \ t \le \frac{m_{itr}}{4} \\ x_{j}^{*t} \times x_{jr_1} \times ES^t \times r & t \le 2 \frac{m_{itr}}{4} \hbox {and} t > \frac{m_{itr}}{4} \end{array}\right. } \end{aligned}$$

Where $x_{j}^{*t}$ is the position $j^{th}$ in the best solution, $\chi$ is the hunting operator, $\nu$ is a sensitive control and equals 0.1, $\gamma _{ij}^t$ is a reduced function, $ES^t$ is the probability ratio known as evolutionary sense, and *r* is a random number, $r \in [0,1]$.

The operators for the hunting process can be computed as follows.

21 $$\begin{aligned} \chi _{ij}^{t}= x_{j}^{*t} \times \delta _{ij} \end{aligned}$$

Where $\delta _{ij}$ is the percentage difference and it can be calculated as shown in Eq. (22).

22 $$\begin{aligned} \delta _{ij}^{t}= \theta +\frac{x_{ij}-avg(x_i)}{x_{j}^{*t} \times (U_j -L_j)+ \varepsilon } \end{aligned}$$

Where $\delta _{ij}^{t}$ is the proportional variance between the present solution’s jth place and the jth position of the best-obtained solution, the solution’s average location is $avg(x_i)$. $\varepsilon$ is a small integer, $\theta$ is a sensitive parameter, and it is equal to 0.1. *i*, *U*, *L* are the upper and lower bounds of the position of *j*. The reduction function $\gamma _{ij}^{t}$ can be computed as shown in Eq. (23).

23 $$\begin{aligned} \gamma _{ij}^{t}= \frac{x_{j}^{*t}-x_{r_2j}}{x_{j}^{*t}+ \varepsilon } \end{aligned}$$

Where $r_2$ is a randomly selected solution in the population, and $avg(x_i)$ is the average location of the solution *i* and it can be computed as shown in Eq. (24).

24 $$\begin{aligned} avg(x_i) = \frac{1}{n}\sum _{j=1}^{n} x_{ij} \end{aligned}$$

The probability ratio called the evolutionary sense ($ES^t$) is obtained using Eq. (25). As the number of iterations increases, its value randomly drops from 2 to -2.

25 $$\begin{aligned} ES^t=2 \times r_3 \times (1-\frac{1}{m_{itr}}) \end{aligned}$$

Where $r_3$ is a random number between -1, 1, $m_{itr}$ is the maximum number of iterations.

#### The exploitation process (hunting).

All of these strategies refer to different intensification techniques that support localized search exploitation. Due to their intensity, crocodile techniques, such as coordination and cooperation in hunting, allow them to approach the target prey more easily than the encircling processes. Thus, the exploitation search reveals the almost perfect solution after maybe a few tries. Furthermore, during this optimization step, an enhanced search near the optimal solution is conducted using the exploitation mechanisms, with a focus on communication between them.

Equation (26) models the two main search strategies used in the RSA exploitation phase, which are (1) hunting coordination and (2) hunting collaboration, to search the search space and find the optimal solution. The hunting coordination method is used to perform the search in this phase when $t \le 3 \frac{m_{itr}}{4}$ and $t > 2 \frac{T}{4}$; when $t \le m_{itr}$ and $t > 3 \frac{T}{4}$, the hunting cooperation approach is used. Be aware that stochastic coefficients are taken into account to produce more dense solutions and to locally exploit the potential locations. The simplest rule possible, which can resemble crocodile hunting techniques, has been used. For the exploitation phase, the following position update equations are suggested as shown in Eq. (26):

26 $$\begin{aligned} x_{ij}^{t+1} = {\left\{ \begin{array}{ll} x_{j}^{*t} \times \delta _{ij} \times r & t \le 3 \frac{m_{itr}}{4} \hbox {and} t> 2 \frac{m_{itr}}{4} \\ x_{j}^{*t} \times -\chi _{ij}^{t} \times \varepsilon - \gamma _{ij}^{t} \times r & \ t \le m_{itr} { and}t > 3 \frac{m_{itr}}{4} \end{array}\right. } \end{aligned}$$

**Fig. 4:**
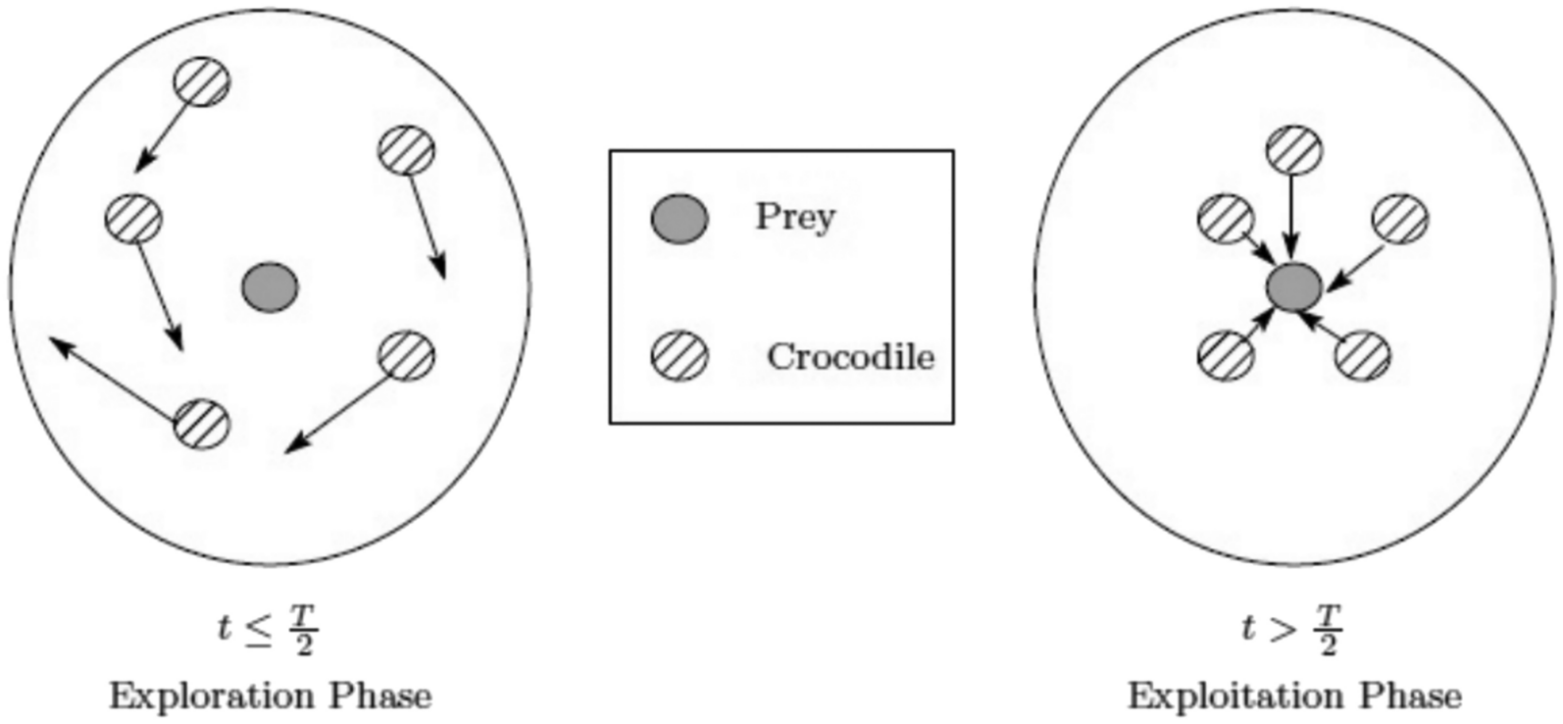
Exploration and exploitation processes.

Figure 4 shows the exploration and exploitation procedures as well as when they are applied.

#### The RSA’s main components.

Algorithm 2 describes the key elements of the RSA.

**Algorithm 2:**
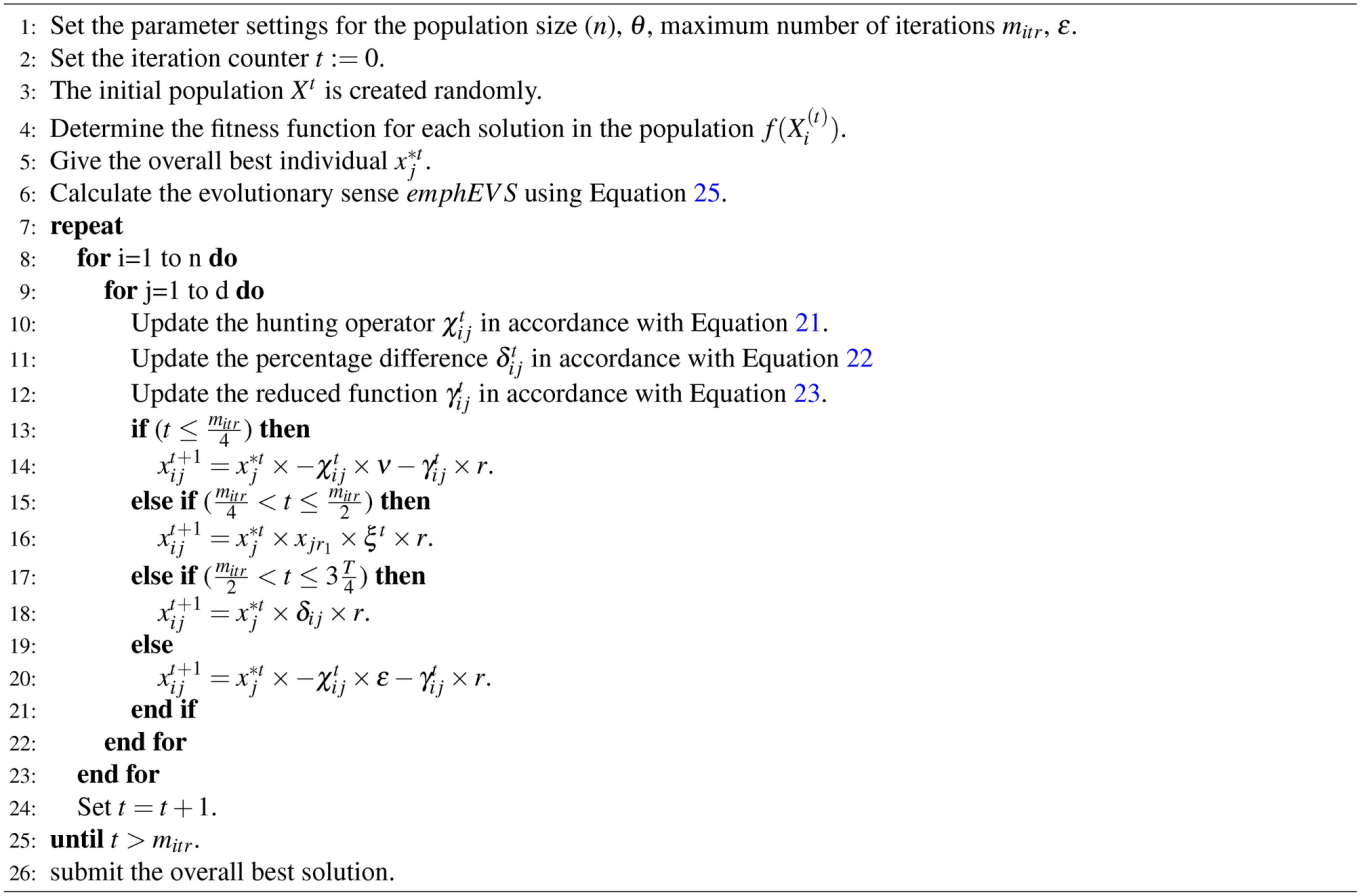
The pseudo-code RSA algorithm.

## The proposed hybrid algorithm

This article combines the traditional gazelle optimization algorithm (GOA) with the traditional reptile search algorithm (RSA) to create a hybrid algorithm. The hybrid gazelle optimization reptile search algorithm (HGORSA) is the name of the suggested algorithm. In the proposed HGORSA, the mathematical models of the exploration and exploitation phases of the traditional GOA have been modified to be more powerful as follows.

### The exploitation (intensification) phase

The exploitation phase in the proposed HGORSA is applied by invoking the hunting operator $\chi _i^{t}$ and the reduction function $\gamma _i^{t}$ in Eq. (29) as follows.

27 $$\begin{aligned} & \chi _i^{t}= X_i^{*t} \times \delta _i \end{aligned}$$

28 $$\begin{aligned} & \gamma _i^{t}= \frac{X_i^{*t}-X_{r_2j}}{X_i^{*t}+ \varepsilon } \end{aligned}$$

Where $X_i^{*t}$ is the overall best solution.

29 $$\begin{aligned} X_i^{t+1} = X_i^{t} + s\cdot R*\cdot X_i^{*t} \times -\chi _i^{t} \times \varepsilon -\gamma _i^{t} \times r. \end{aligned}$$

Where $X_i^{t+1}$ is a solution in the next iteration, *s* represents the speed with which the gazelles graze and the vector *R* contains uniform random numbers in [0,1].

#### The exploration (diversification) phase

The exploration phase in the proposed HGORSA can be defined as shown in Eqs. (30), (31) by modifying Eqs. (15), (16) as follows.

30 $$\begin{aligned} X_i^{t+1} = X_i^{t} + MS\cdot \mu \cdot R *\cdot X_i^{*t} \times -\chi _i^t \times \nu - \gamma _i^{t} \times r. \end{aligned}$$

Where *MS* is the maximum speed a gazelle can go, $\mu$ is a change of direction, $\mu \in [-1,1]$, $\nu$ is a sensitive operator, and *r* is a random number. Equation (16) illustrates the mathematical model for the predator’s pursuit of the gazelle and it can be modified as shown in Eq. (31).

31 $$\begin{aligned} X_i^{t+1} = X_i^{t} + MS\cdot \mu \cdot CF *\cdot x_{j}^{*t} \times x_{jr_1} \times ES^t \times r. \end{aligned}$$

Where $CF = (1-\frac{itr}{m_{itr}})^{(2\frac{itr}{m_{itr}})}$ is the cumulative effect of predator, $m_{itr}$ is the maximum number of iterations, *r* is a random number between 0,1.

### The pseudo-code of the proposed HGORSA

**Fig. 5:**
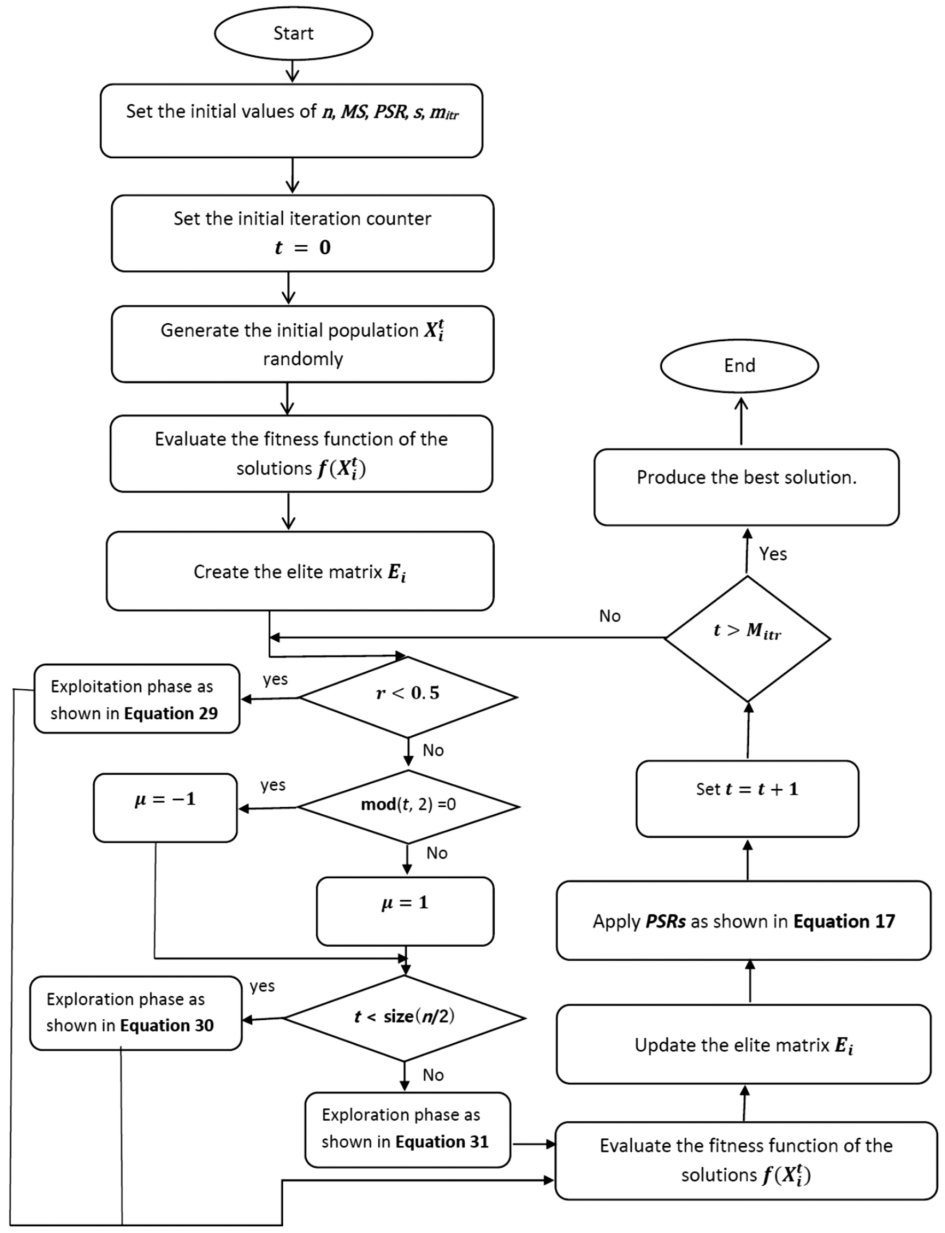
Flowchart of the HGORSA.

The main steps of the proposed HGORSA are reported in Algorithm 3 and Fig. 5 as follows.

**Algorithm 3:**
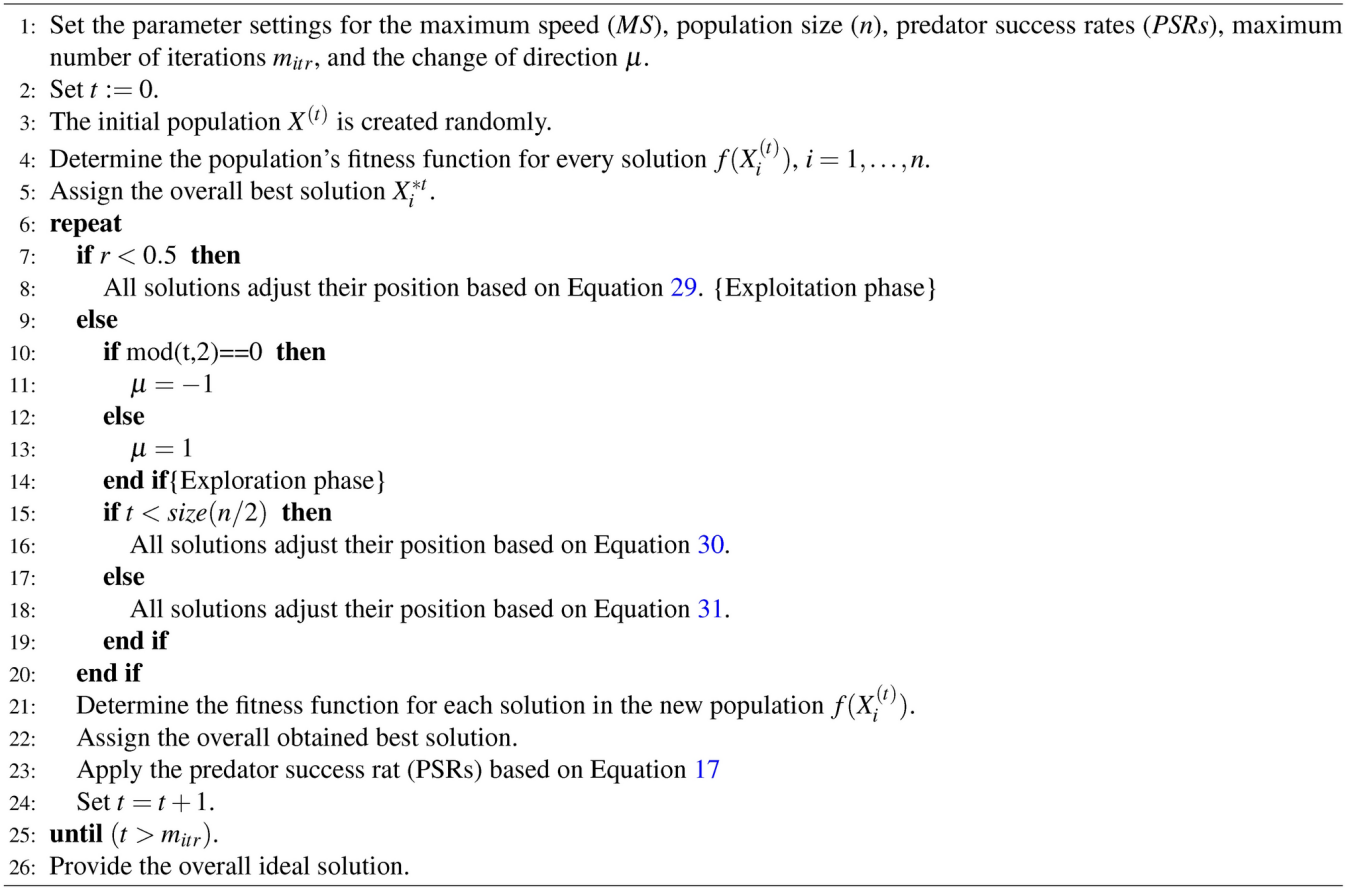
The HGORSA algorithm.

The HGORSA steps can be summed up as follows.

- **Initialization of the parameters.** The initial setting of the parameters is assigned such as maximum speed (*MS*), population size (*n*), predator success rates (*PSRs*), the maximum number of iterations $m_{itr}$, and the change of direction operator $\mu$.
- **Initialization of the Iteration counter.** The initialization of the iteration counter is set to $t:=0$.
- **The creation of the initial population.** The population’s solutions are produced at random using $X^{(t)}$.
- **The fitness function calculation.** The fitness function $f(X_i^{(t)})$ is determined for each solution in the population.
- **The assignment of the best solution** Overall the best solution $X_i^{*t}$ is assigned.
- **Position update for the solutions.** Depending on the value of *r*, the algorithm switches between the exploitation and the exploration phases.
- **The exploitation phase.** The exploitation phase starts by updating all the solutions based on Eq. (29).
- **The exploration phase.** The exploration phase starts by updating all solutions based on Eqs. (30) and (31).
- **The evaluation of new solutions.** The fitness function $f(X_i^{(t)})$ is determined for each solution in the population.
- **The update of the best solution** An updated elite matrix is produced along with the assignment of the new global best solution.
- **The predator success rates (PSRs)** The predator success rates (PSRs) is applied based on Eq. (17).
- **Increase in iteration Counter.** The iteration number counter is raised to $t=t+1$.
- **Satisfaction of termination criteria.** The procedures are repeated until the allotted number of times, $m_{itr}$, has been reached.
- **The global best solution.** The optimal solution is generated.

### The computational complexity analysis of the proposed HGORSA

The computational complexity of the proposed HGORSA is determined by three operations: generating the initial population *n*, calculating the fitness function for all individuals, and updating the individuals, as described below.

- **The generation of the initial population** The time complexity of generating the initial population is $O(n \times D)$, where *D* is the dimension of individuals.
- **The fitness function calculation of the population** The time complexity of calculating the fitness function of all individuals in the population is $O (m_{itr} \times n \times D)$, where $m_{itr}$ is the maximum number of iterations.
- **The individual updating process** The time complexity of updating all individuals in the population is $O (m_{itr} \times n \times D)$.
- **The total time complexity of the proposed HGORSA** The total time complexity of the proposed algorithm is $O(n \times D) + O (m_{itr} \times n \times D) + O (m_{itr} \times n \times D)$ = $O(nD(1+2m_{itr}))$.

## Numerical results and discussion

The simulation and performance measurement experiments for all algorithms are coded by MATLAB R2020a.The simulation was carried out using a laptop with the following configurations which are an Intel Core i7-6820HQ, 8 GB of RAM, and the Microsoft Windows operating system. A set of performance criteria is used to assess the suggested HGORSA’s performance, which are the worst, Avg, Std, and best values. In addition, five standard performance metrics are measured that include energy consumption, network throughput, number of dead nodes, network lifetime, and stability period. Moreover, to get stable statistical results that prove the robustness and precision of meta-heuristic algorithms, the trial was run 20 times along with the proposed algorithm and all comparative algorithms. To display and render the results, the average of these occurrence times is used after 5000 iterations each time.

### Simulation setup

**Fig. 6:**
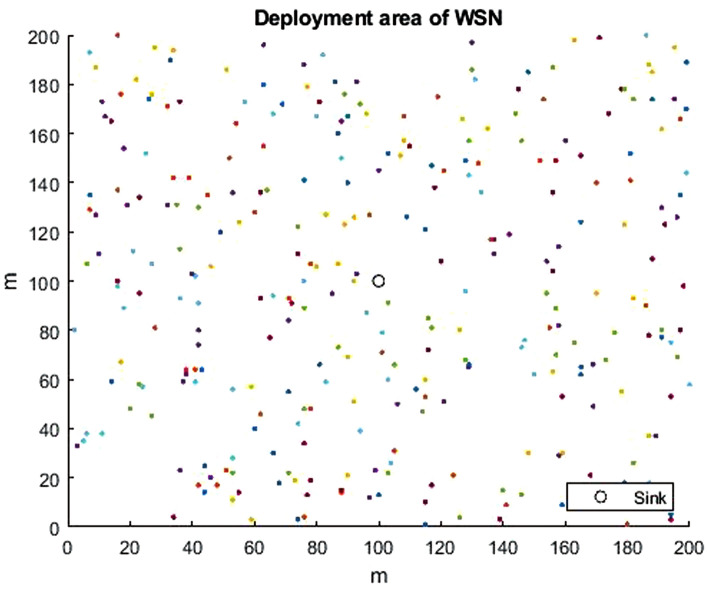
WSN topology of 300 SNs and the sink is at (100, 100).

**Fig. 7:**
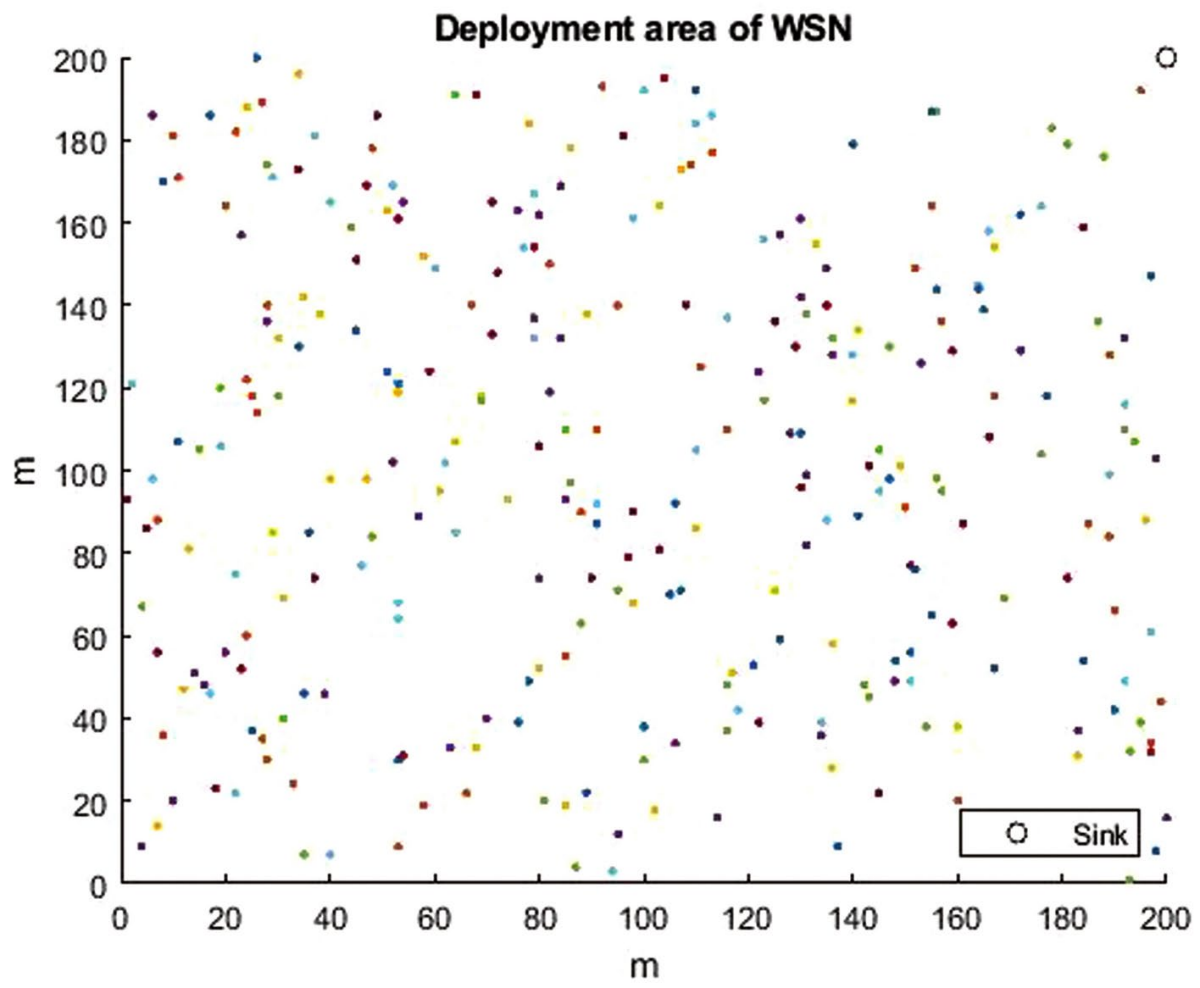
WSN topology of 300 SNs and the sink is at (200, 200).

**Fig. 8:**
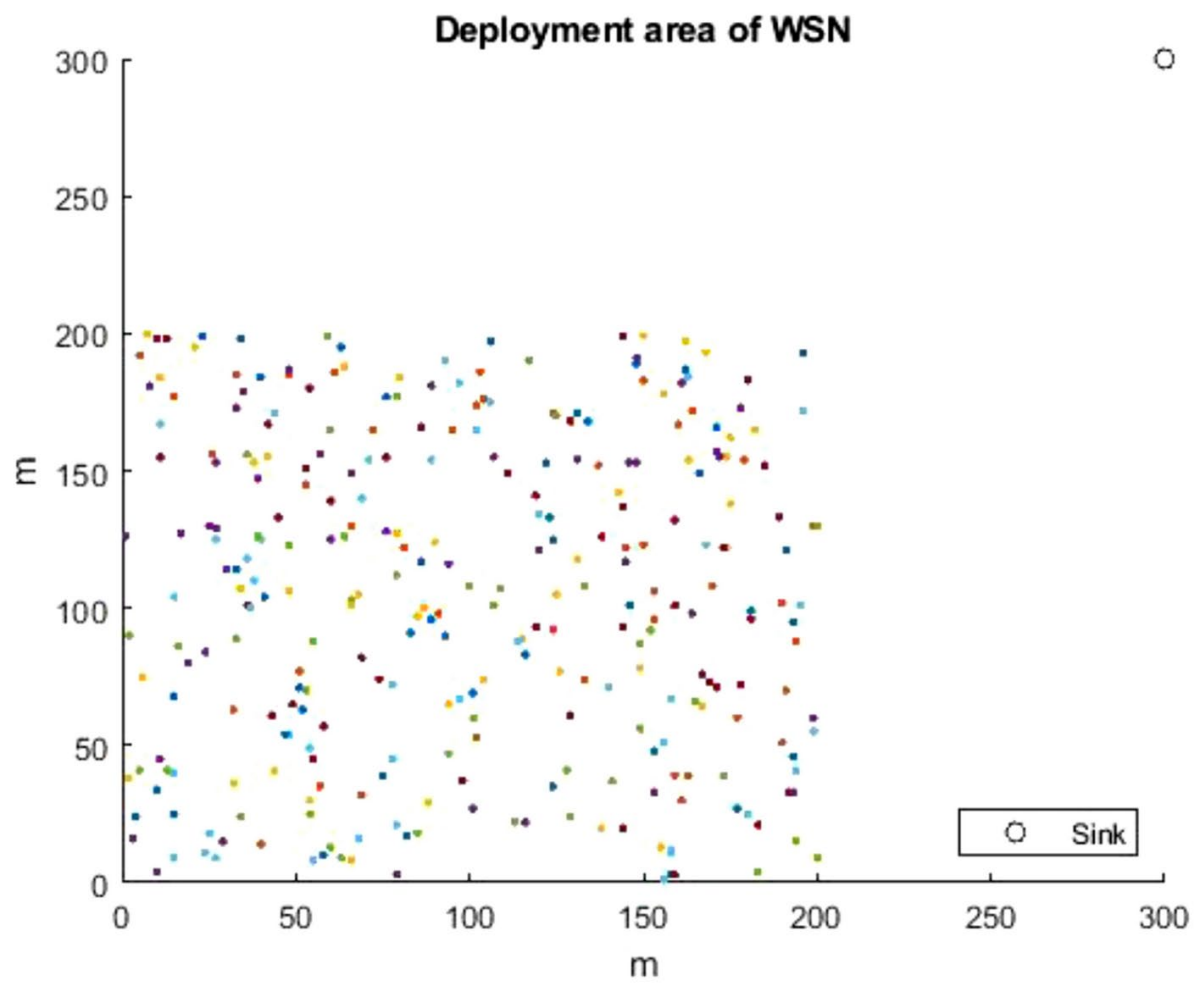
WSN topology of 300 SNs and the sink is at (300, 300).

The network configuration is done through the setup phase. The WSN consisted of 300 homogeneous random SNs in a deployment area of $(200 m \times 200 m)$. The simulations are performed allowing the sink to be transferred to three different locations, which are the center of the field (100, 100), corner of the field (200, 200), and field outside (300, 300) as indicated in Figs. 6, 7, 8. More parameter values are listed in Table 3, which have been proposed by Heintzelman et al. in^50^. The parameters used for the proposed HGOARSA are reported in Table 4. Since *n* refers to the number of the gazelle population. *D* indicates dimension of the problem equal to 30. The lower and upper values of the search domain were donated by LB and UB. $m_{itr}$ is the iteration maximum number, and it is equal to 5000. Whereas $\alpha$ is a parameter that controls the parameters of energy and distance. *PSRs* is set to 0.34. Here, *S* is equal to 0.88. Both $\alpha _1$ and $\beta _1$ are sensitive parameters to control exploration accuracy and both to 0.1. Finally, by following subsections, the performance evaluation of all the algorithm results will be explored and analyzed.

**Table 3:** Parameters used in the network implementation.

Parameter	Explanation	Value
$E_{TX}$	Energy loss when transmitting data.	50nJ/bit
$E_{RX}$	Energy loss when receiving data.	50nJ/bit
$\varepsilon _{fs}$	Amplification energy of free space module.	10PJ/bit/m²
$\varepsilon _{mp}$	Amplification energy of multiple-path module.	0.003nJ/bit
$E_{DA}$	Energy for data aggregation.	5nJ/bit

**Table 4:** HGORSA parameters.

Parameter	Explanation	Value
*n*	Population size	300
*D*	Problem dimension.	30
*LB*	Lower limit.	1
*UB*	Upper limit.	200
*PSRs*	The predator success rate.	0.34
*S*	The speed of gazelles when grazing.	0.88
$\beta _1$	Control parameter.	0.1
$\alpha _1$	Control parameter.	0.1
$\alpha$	Control parameter.	0.3
$m_{itr}$	No. of maximum iterations.	5000

### The proposed HGORSA evaluations

To evaluate the robustness of the HGORSA algorithm, a multitude of experiments are conducted to compare the algorithm suggested by six state-of-the-art metahuirstic algorithms, exploiting five standard evaluation metrics, which are energy consumption, dead node number, network lifetime, network throughput, and stability period with different network size for SNs. The simulation process begins with the main experiment, in which the number of SNs is equal to 300. The HGORSA algorithm is tested when the number of SNs is equal to 50 and 500 as an additional experiment. All simulation and statistical outputs are highlighted in the subsequent sections. The performance metrics used are defined as the following:

- **Energy consumption :** It is a metric widely used to evaluate the efficiency of the clustering algorithm. It is measured in joules (Js) and represents the total expected energy over the entire lifetime of the network. The algorithm’s efficiency can be estimated on the basis of the total amount of energy that is spent.
- **Dead nodes number:** This means several dead or inactive nodes at the end of the entire network lifetime.
- **Network lifetime:** To analyze the lifetime of the network, the HND metric is established. This metric indicates the number of iterations in which the half nodes die.
- **Network throughput: ** This measurement verifies the total number of packets that the receiver(the sink) acknowledges throughout the lifetime of the network. In networking, it is a valuable parameter to measure the efficiency of a routing algorithm.
- **Stability period:** To determine the stability period of the network, the first node death metric (FND) has been used. FND measures which iteration number the first node death occurs in the network. It is considered an important factor for network reliability. The longer stability period of the network guarantees superb network reliability.

### Performance evaluation of proposed HGORSA and two its components in terms of energy consumption

**Fig. 9:**
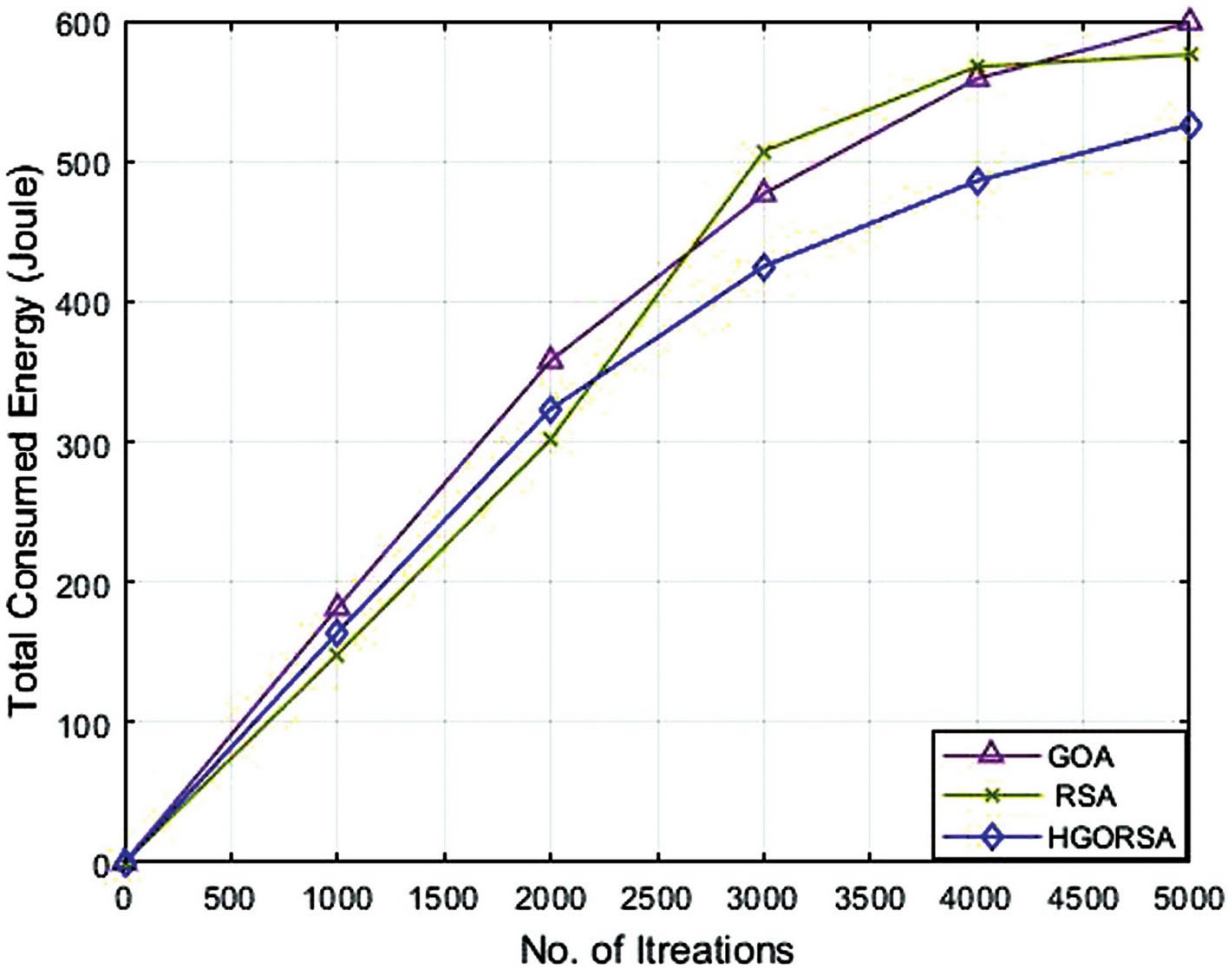
Energy consumption of proposed HGORSA, GOA and RSA versus 5000 iterations and sink is at (100, 100).

**Fig. 10:**
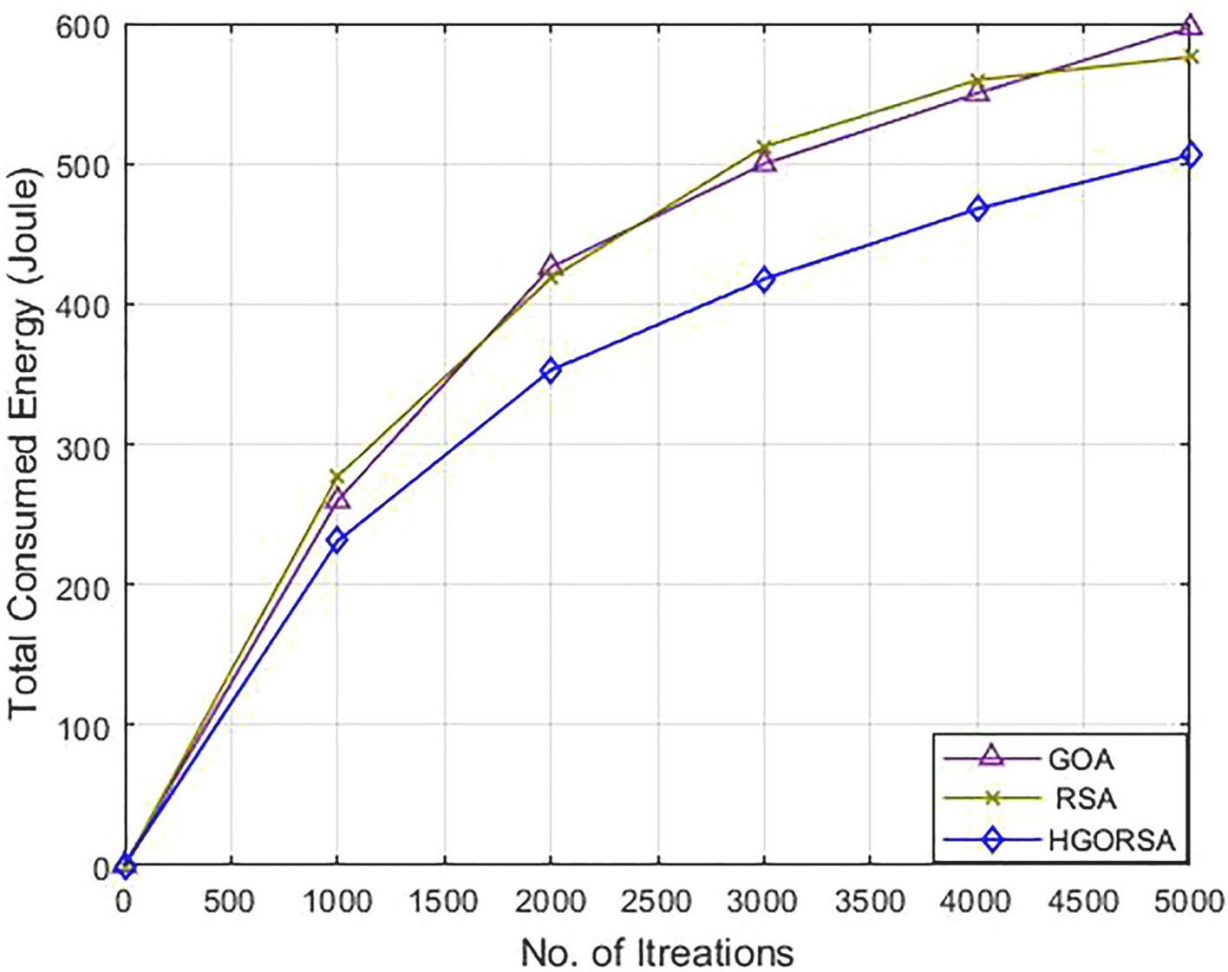
Energy consumption of proposed HGORSA, GOA and RSA versus 5000 iterations and sink is at (200, 200).

**Fig. 11:**
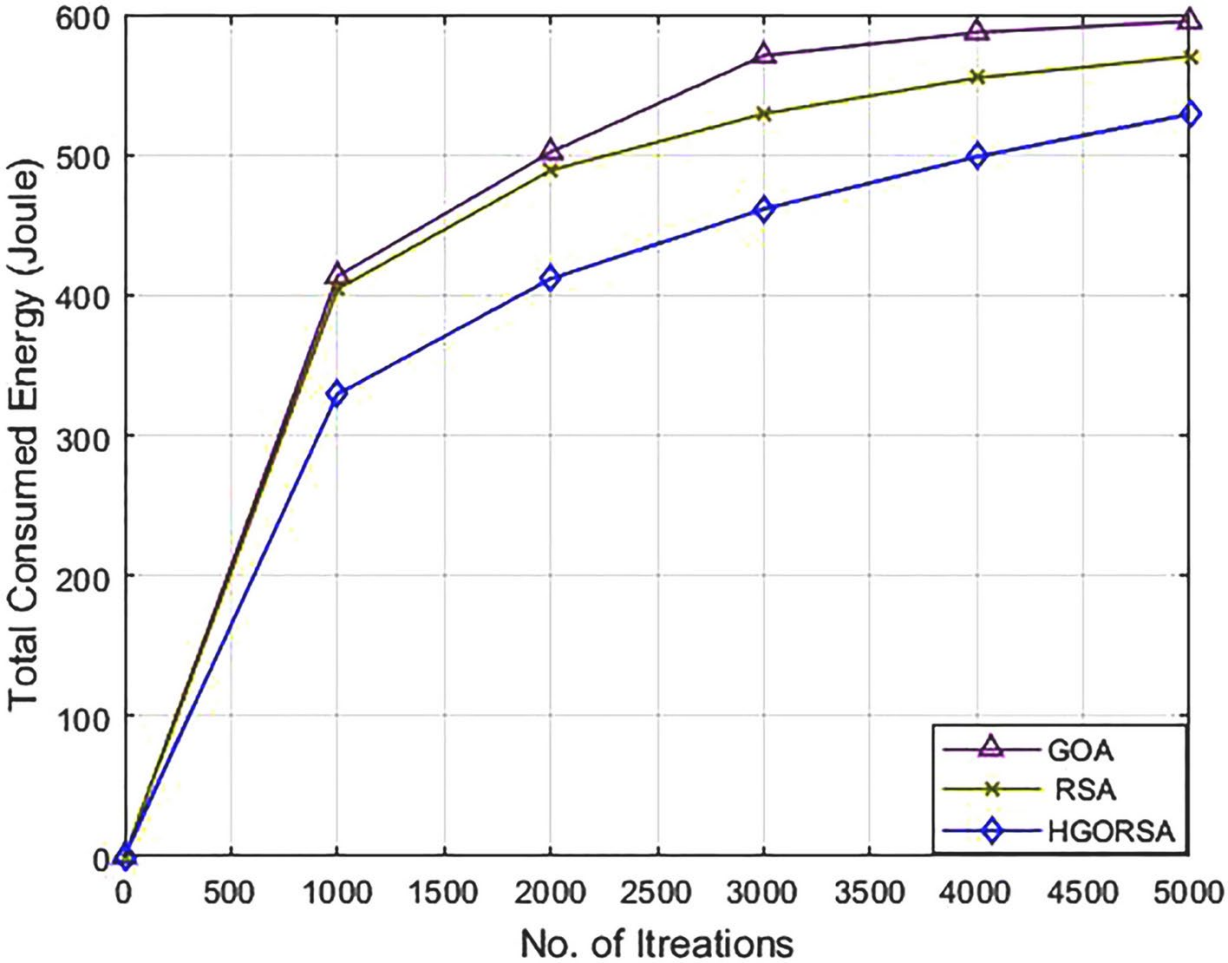
Energy consumption of proposed HGORSA, GOA, and RSA versus 5000 iterations and sink is at (300, 300).

The proposed HGORSA perfectly improves network energy consumption compared to both of its components, which are: GOA and RSA individually with three cases for the sink position. The first position is a central position within the sensing field at $(100\times 100)$, the second is in the corner $(200\times 200)$, and the third sink position is out of the sensing field $(300\times 300)$ as shown in Figs. 9, 10, 11. The total energy consumption of HGORSA for the network is the lowest; hence, the network performance is higher when the sink is centrally located. When the sink is located outside the sensing field, the total energy consumption of the network is higher and the network performance is lower.

### Performance evaluation of proposed HGORSA and two of its components in terms of dead nodes number

**Fig. 12:**
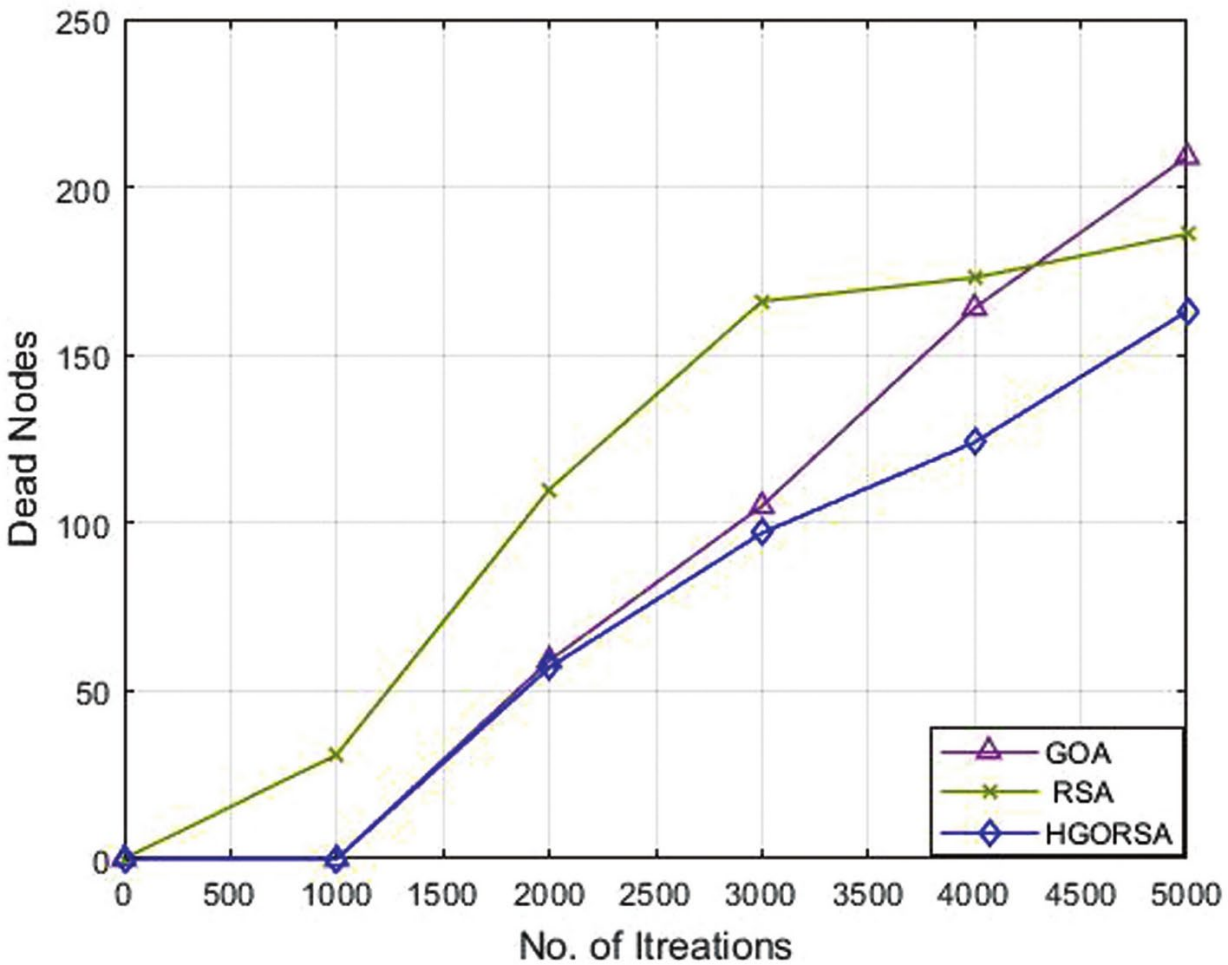
Dead nodes number for HGORSA, GOA, and RSA and the sink is in field centre.

**Fig. 13:**
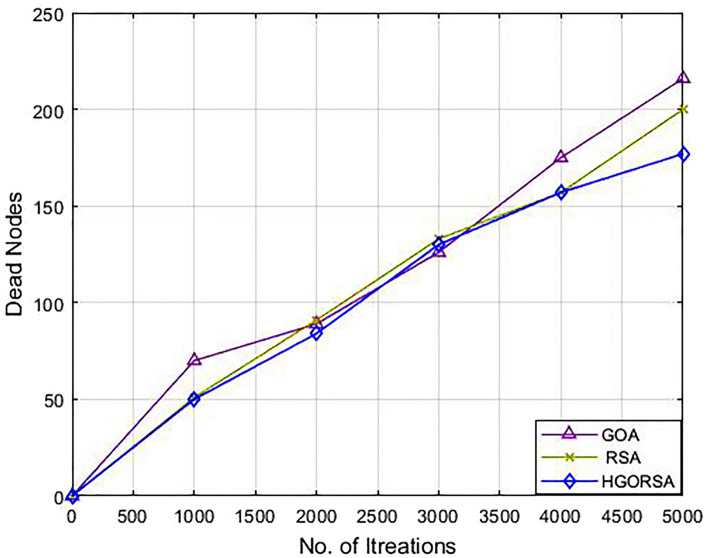
Dead nodes number for HGORSA, GOA, and RSA and the sink is in field right corner.

**Fig. 14:**
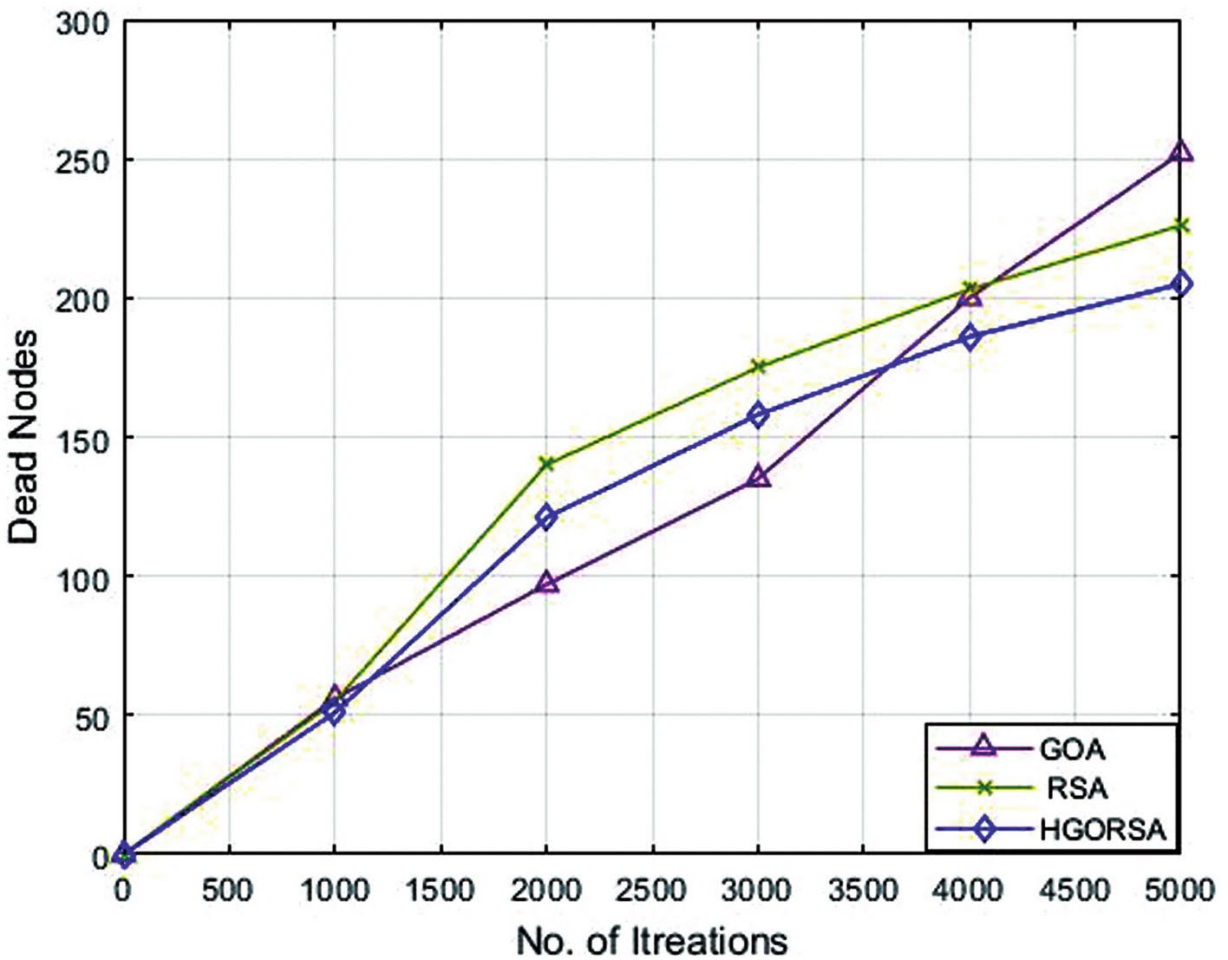
Dead nodes number for HGORSA, GOA, and RSA and the sink is in the field outside.

Plethora of indicators are used to measure the performance of WSNs, among them computing the number of dead nodes. If there are fewer dead nodes, the network has a long lifetime. Simulation experiments prove that the proposed HGORSA can also utilize the rate of live nodes. This means reducing the rate of dead nodes and maximizing the rate of alive nodes. The relationship between iterations and dead nodes is plotted in Figs. 12, 13, 14 for HGORSA, GOA, and RSA.

### Performance evaluation of proposed HGORSA and other meta-heuristic algorithms in terms of energy consumption

The simulation process begins with the main experiment, in which the number of SNs is equal to 300 and the proposed HGORSA and other existing algorithms are tested under similar conditions for performance comparisons. HGORSA is compared with six state-of-the-art meta-heuristic algorithms such as particle swarm optimization (PSO)^51^, greywolf optimizer (GWO)^52^, sperm swarm optimization (SSO)^53^, Chernobyl disaster optimizer (CDO)^54^, gazelle optimization algorithm (GOA) ,^22^ and reptile search algorithm (RSA)^23^ in three sink position scenarios. Here, the energy consumption of HGORSA and the aforementioned algorithms is compared by some statistical measures such as Std, Avg, the worst, and the best value reported in Tables 5, 6 and 7.

**Table 5:** Best, worst, Avg, and Std values of energy consumption when the sink is at (100, 100).

Algorithms	Best	Worst	Avg	Std
PSO	552.70	589.40	576.26	17.47
GWO	545.30	600.50	574.83	15.71
SSO	542.94	600.36	572.41	15.63
CDO	540.48	600.11	562.73	23.86
GOA	530.29	597.38	542.09	27.60
RSA	518.30	586.83	533.90	26.42
HGORSA	**473.24**	**552.12**	**517.58**	**14.57**

**Table 6:** Best, worst, Avg, and Std values of energy consumption when the sink is at (200, 200).

Algorithms	Best	Worst	Avg	Std
PSO	566.40	596.40	577.26	13.89
GWO	547.30	590.60	574.86	16.00
SSO	561.78	601.12	566.61	16.41
CDO	531.45	596.96	561.13	26.07
GOA	533.90	598.20	551.42	32.48
RSA	518.30	586.83	542.09	26.42
HGORSA	**501.55**	**566.12**	**521.19**	**11.45**

**Table 7:** Best, worst, Avg, and Std values of energy consumption when the sink is at (300, 300).

Algorithms	Best	Worst	Avg	Std
PSO	595.70	602.20	601.40	2.42
GWO	587.22	602.30	599.10	6.83
SSO	584.87	601.12	586.24	8.62
CDO	542.78	600.37	581.62	23.97
GOA	530.64	601.18	579.99	26.67
RSA	522.65	594.95	545.49	27.14
HGORSA	**513.58**	**564.44**	**531.26**	**7.37**

**Fig. 15:**
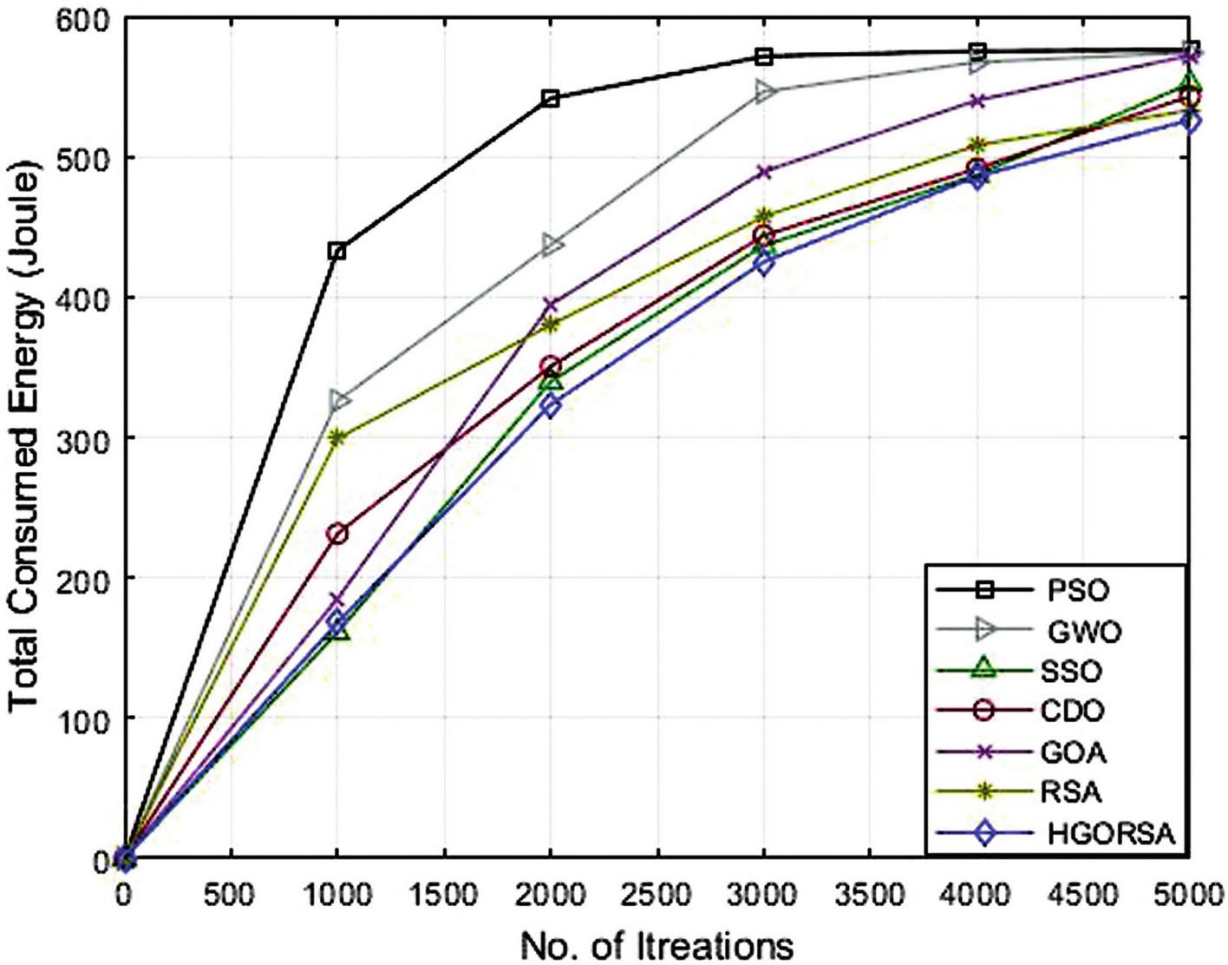
Energy consumption of proposed HGORSA and other meta-heuristic algorithms verses 5000 iterations and sink is at (100, 100).

**Fig. 16:**
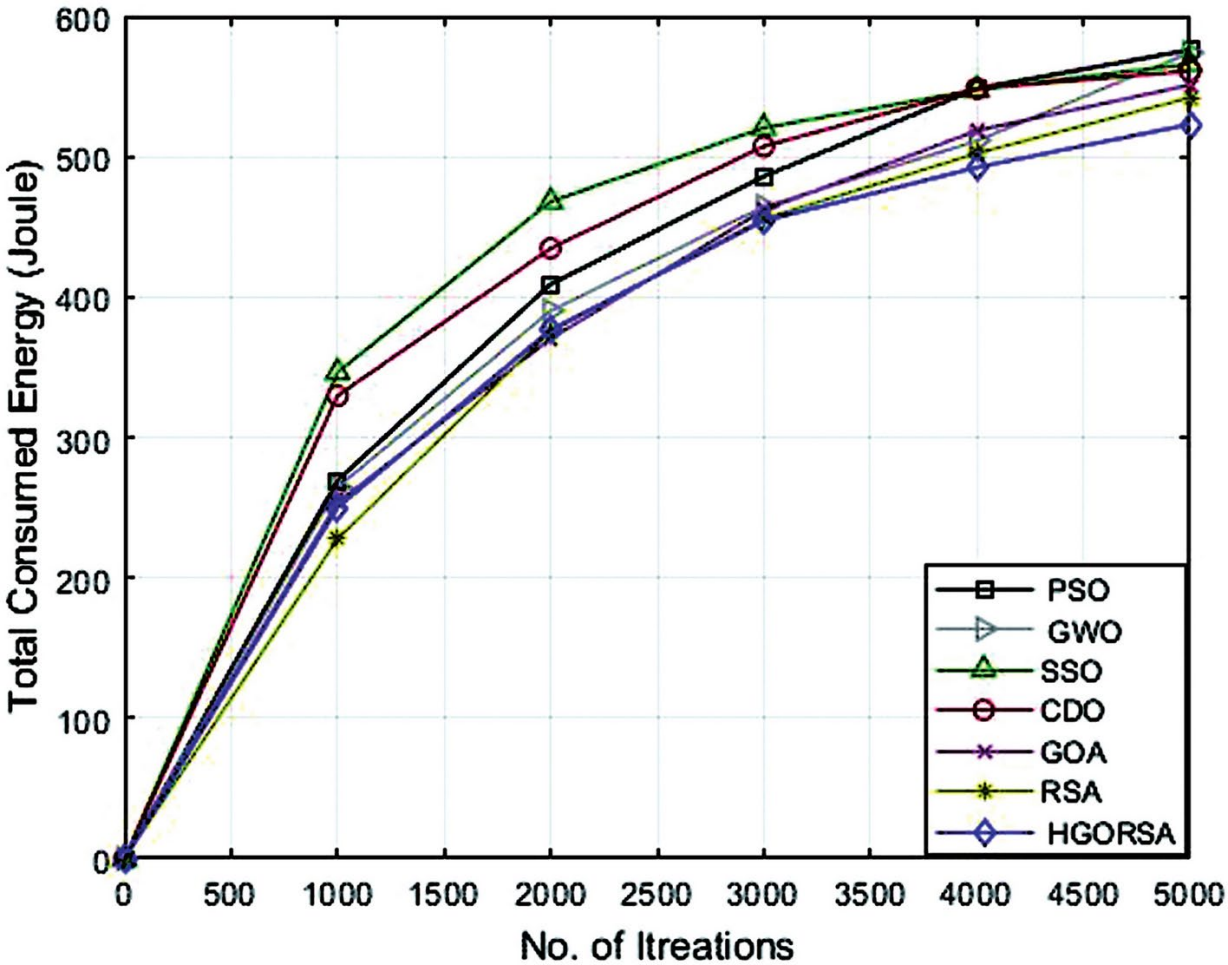
Energy consumption of proposed HGORSA and other meta-heuristic algorithms verses 5000 iterations and sink is at (200, 200).

**Fig. 17:**
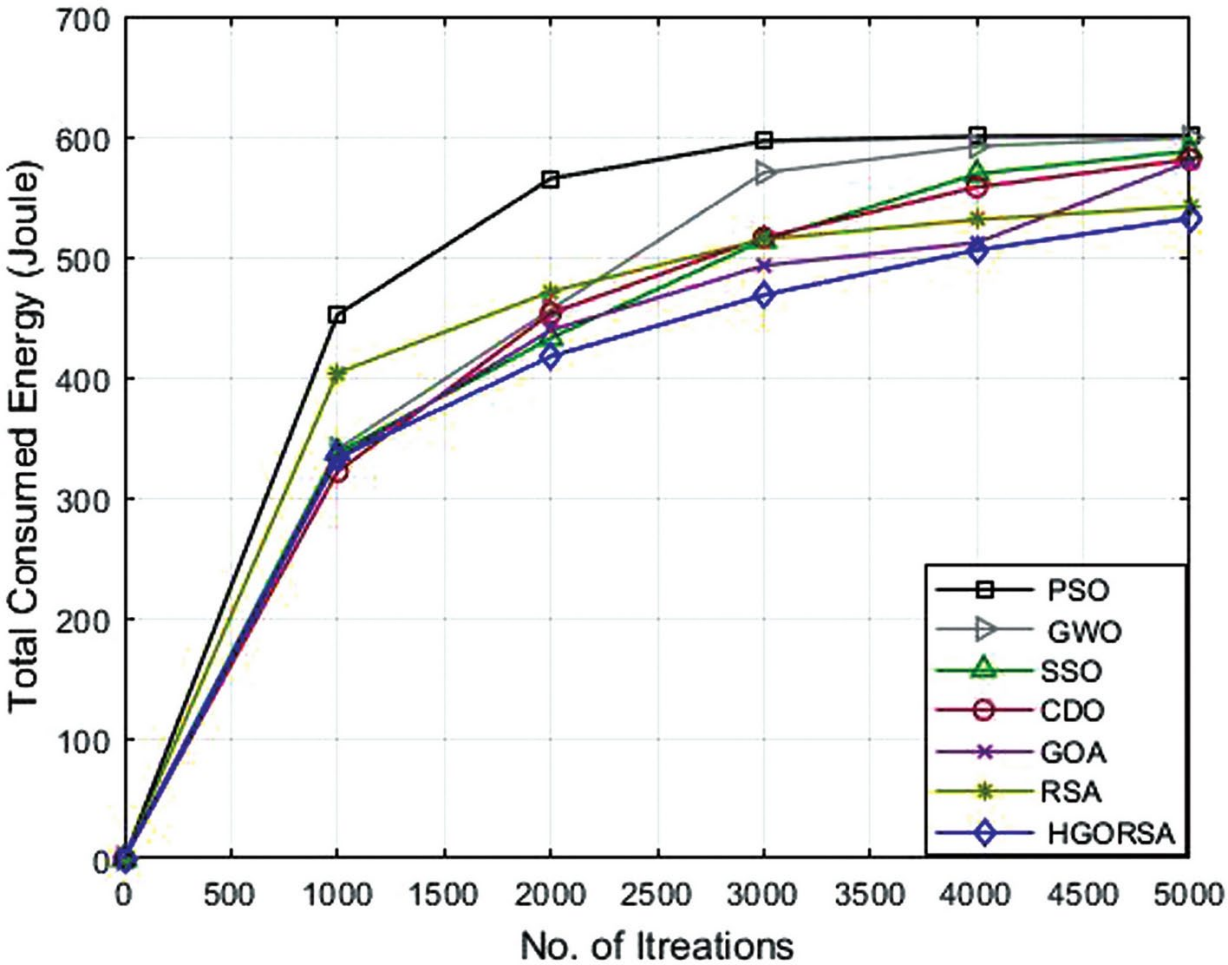
Energy consumption of proposed HGORSA and other meta-heuristic algorithms verses 5000 iterations and sink is at (300, 300).

Figs. 15, 16, and 17 give the convergence graph of the proposed algorithm and the other meta-heuristic algorithms for the analysis of the consumed total energy for the different aforementioned algorithms with three different sink positions(100, 100), (200, 200) and (300, 300), respectively. Observe that the proposed HGORSA consumes less energy than other existing algorithms.

### Performance evaluation of proposed HGORSA and other meta-heuristic algorithms in terms of dead nodes number

**Fig. 18:**
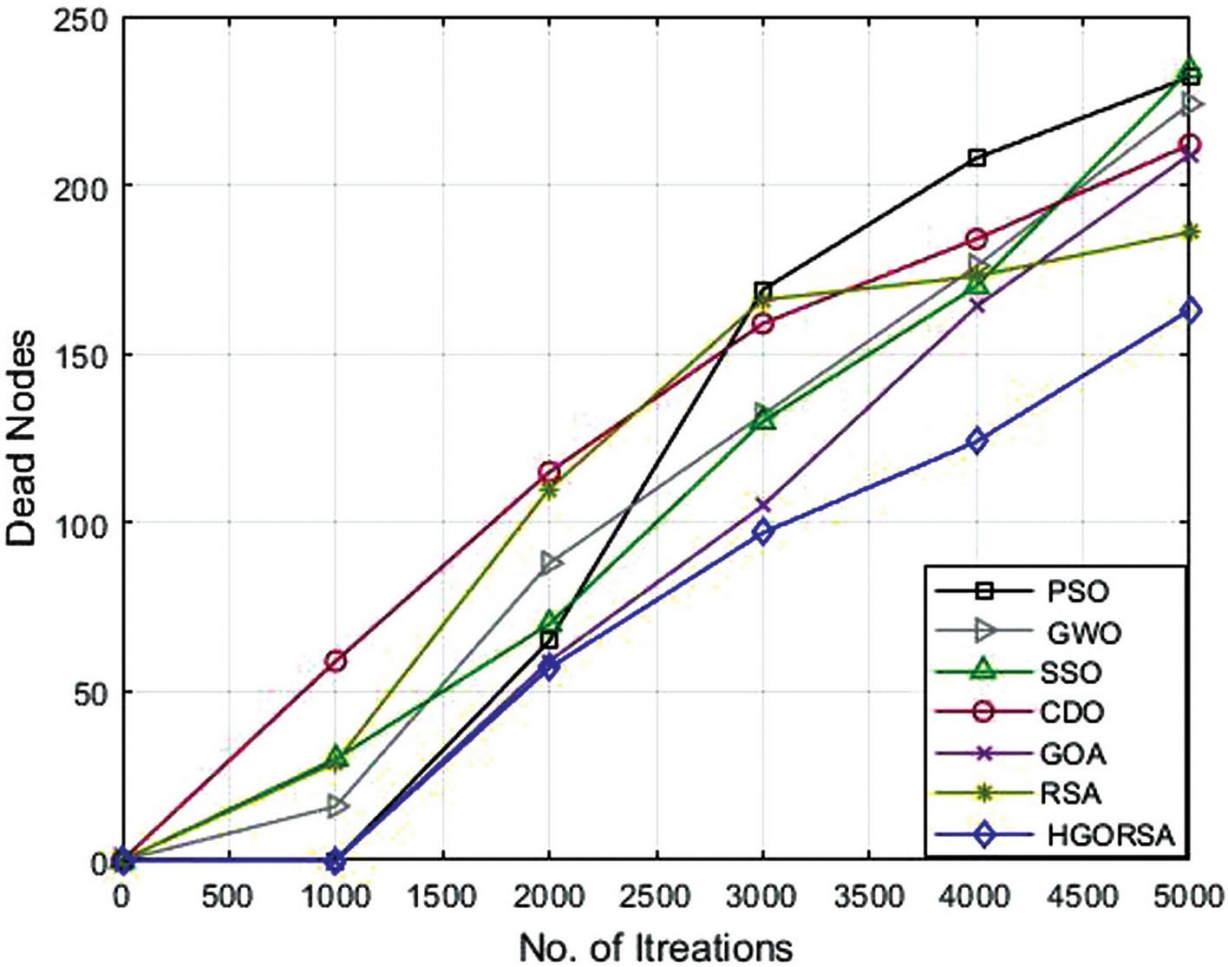
Dead nodes number of HGORSA and other meta-heuristic algorithms when the sink is at (100, 100).

**Fig. 19:**
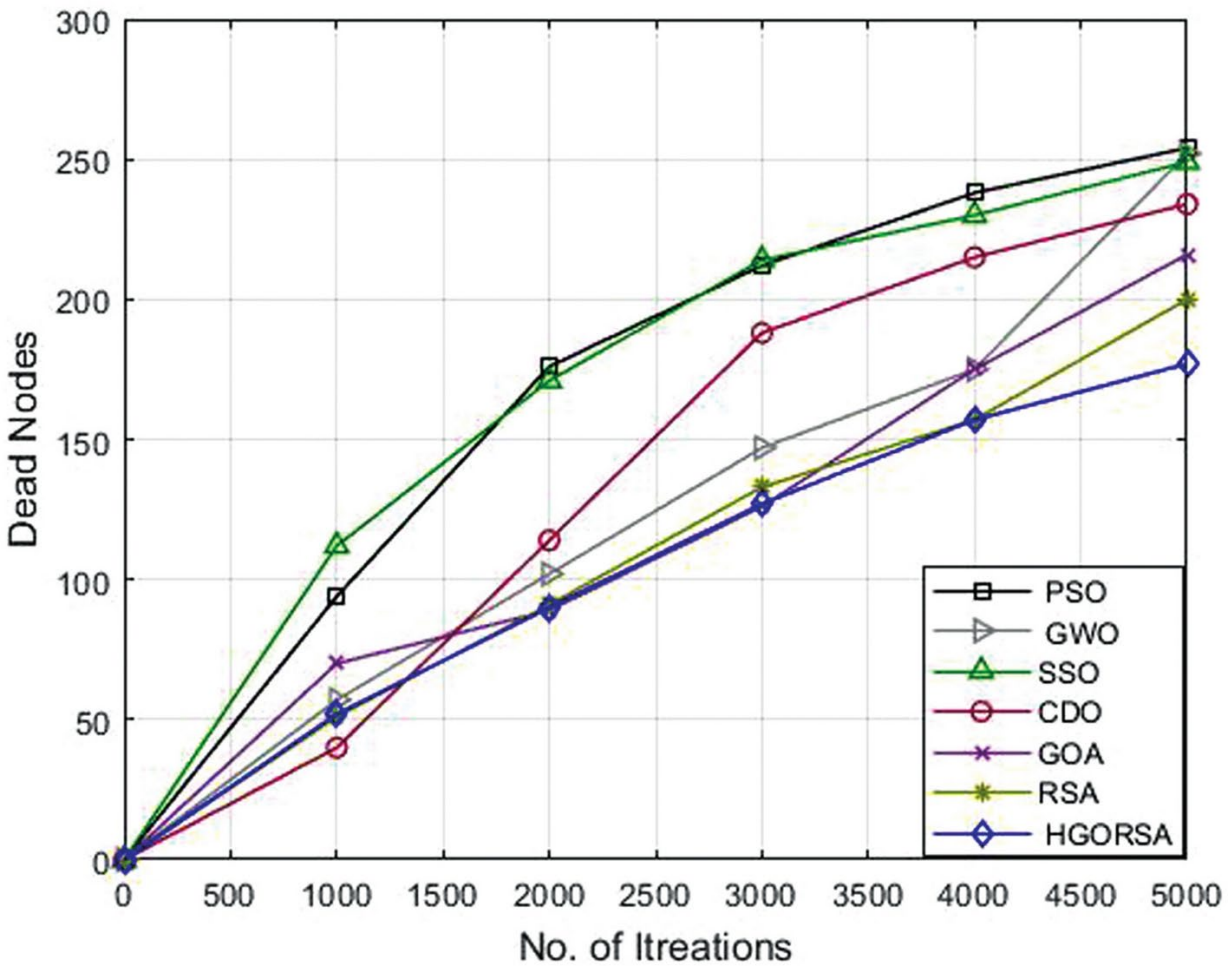
Dead nodes number of HGORSA and other meta-heuristic algorithms when the sink is at (200, 200).

**Fig. 20:**
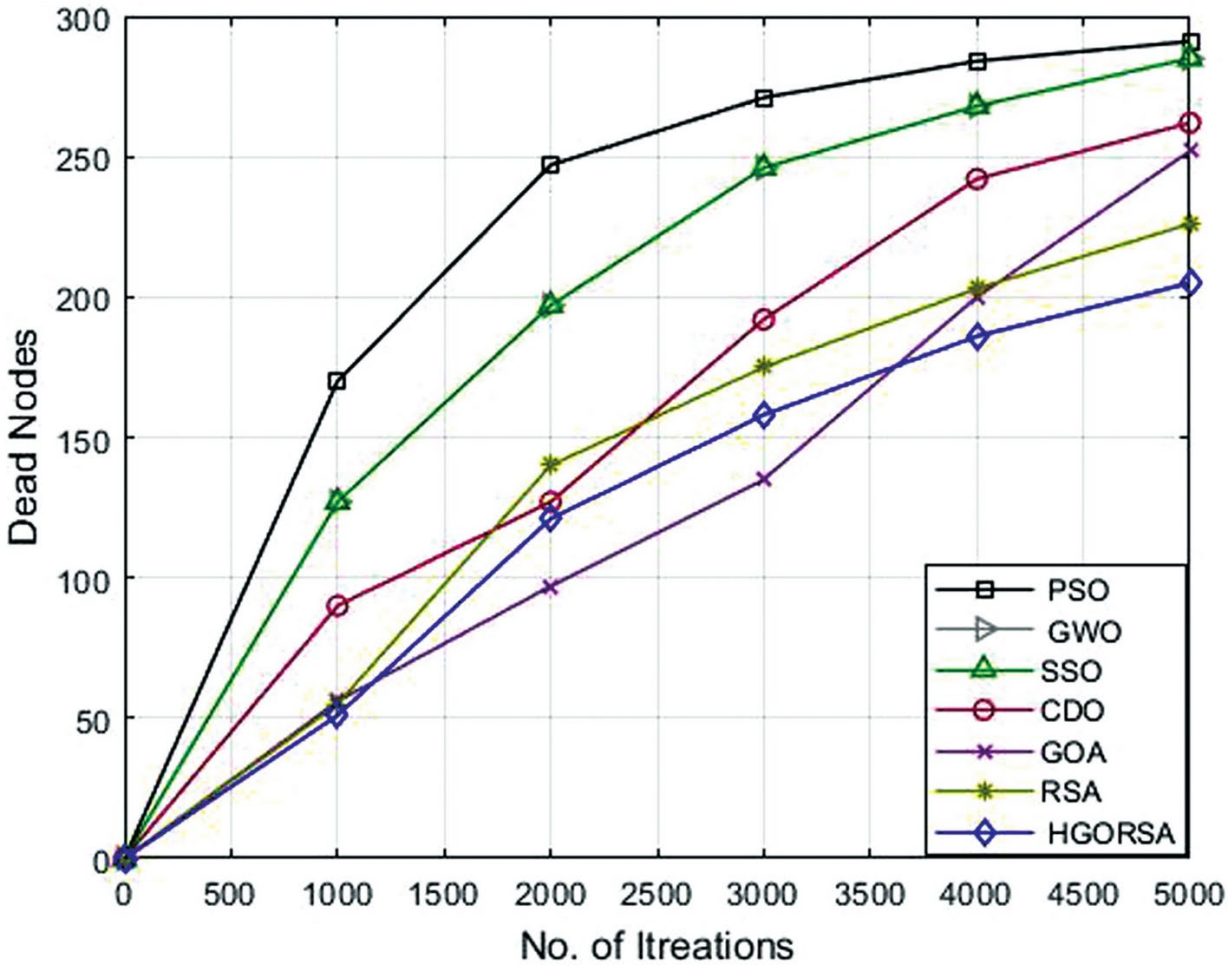
Dead nodes number of HGORSA and other meta-heuristic algorithms when the sink is at (300, 300).

The number of dead nodes has been measured throughout the life of the network for the proposed and other existing algorithms. From simulation outputs, PSO has the highest number of dead nodes (232, 254, 291), while HGORSA has the smaller number of dead nodes (163, 187, 205) at sink positions of (100, 100), (200, 200) and (300, 300). More details of the results of the remaining algorithms are given in Table 8. The conclusion says that HGORSA achieves progress in fewer dead node numbers by 30. 3%, 29. 7%, 28. 9%, 24. 3%, 18% and 11. 5%, respectively. The simulation graphs for the analysis of the dead nodes are shown in Figs. 18, 19, 20

**Table 8:** Dead nodes number for proposed HGORSA and other meta-heuristic algorithms when the sink is at three different positions.

Algorithms	Field center	Field right corner	Field outside
PSO	232	254	291
GWO	224	252	285
SSO	234	249	285
CDO	212	234	262
GOA	209	216	252
RSA	186	200	226
HGORSA	**163**	**177**	**205**

### Performance evaluation of proposed HGORSA and other meta-heuristic algorithms in terms of network lifetime (HND)

**Fig. 21:**
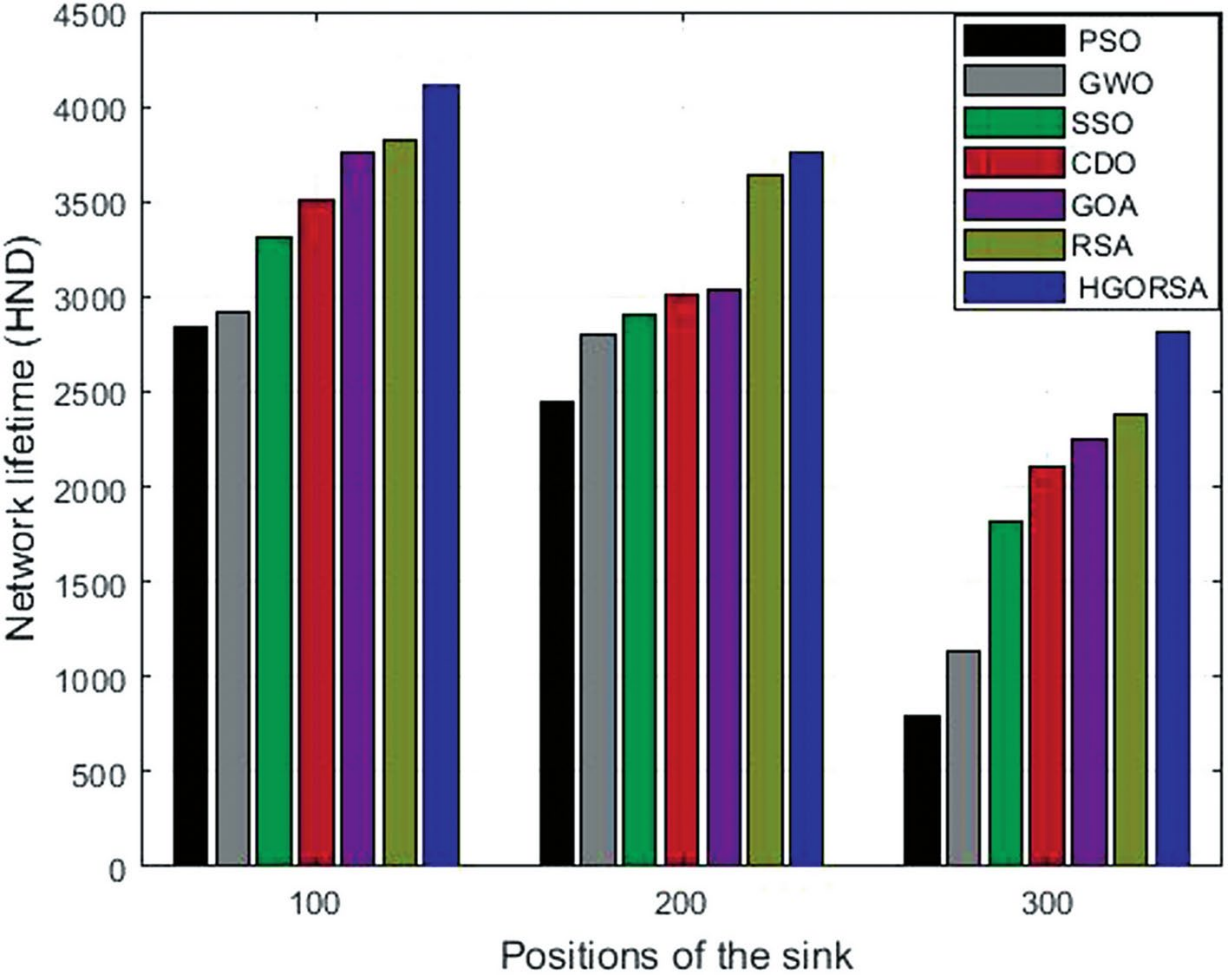
Network lifetime (HND) for HGORSA and other meta-heuristic algorithms at different positions of the sink.

Simulation results show that HGORSA improves the lifetime of the network represented by the HND metric for 44.5%, 40.8%, 23.8%, 16.8%, 9.3%, and 7.2%, respectively than PSO, GWO, SSO, CDO, GOA, and RSA as demonstrated in Table 9. When the position of the sink is (100,100), for PSO, the HND metric takes place for about 2841 iterations, while the half-node death for HGORSA takes place for about 4107 iterations. This means that PSO has the shortest network lifetime (HND) and HGORSA has the longest network lifetime. Figure 21 demonstrated that HGORSA makes improvements compared to meta-heuristic algorithms, and this indicates extended network lifetime for the HGORSA algorithm at three sink positions (100, 100),(200, 200) and (300, 300).

**Table 9:** HND for HGORSA and other meta-heuristic algorithms when the sink is at three different positions.

Algorithms	Field center	Field right corner	Field outside
PSO	2841	2449	795
GWO	2916	2805	1136
SSO	3317	2901	1815
CDO	3516	3012	2105
GOA	3757	3041	1815
RSA	3830	3636	2375
HGORSA	**4107**	**3763**	**2814**

### Performance evaluation of proposed HGORSA and other meta-heuristic algorithms in terms of network throughput

**Fig. 22:**
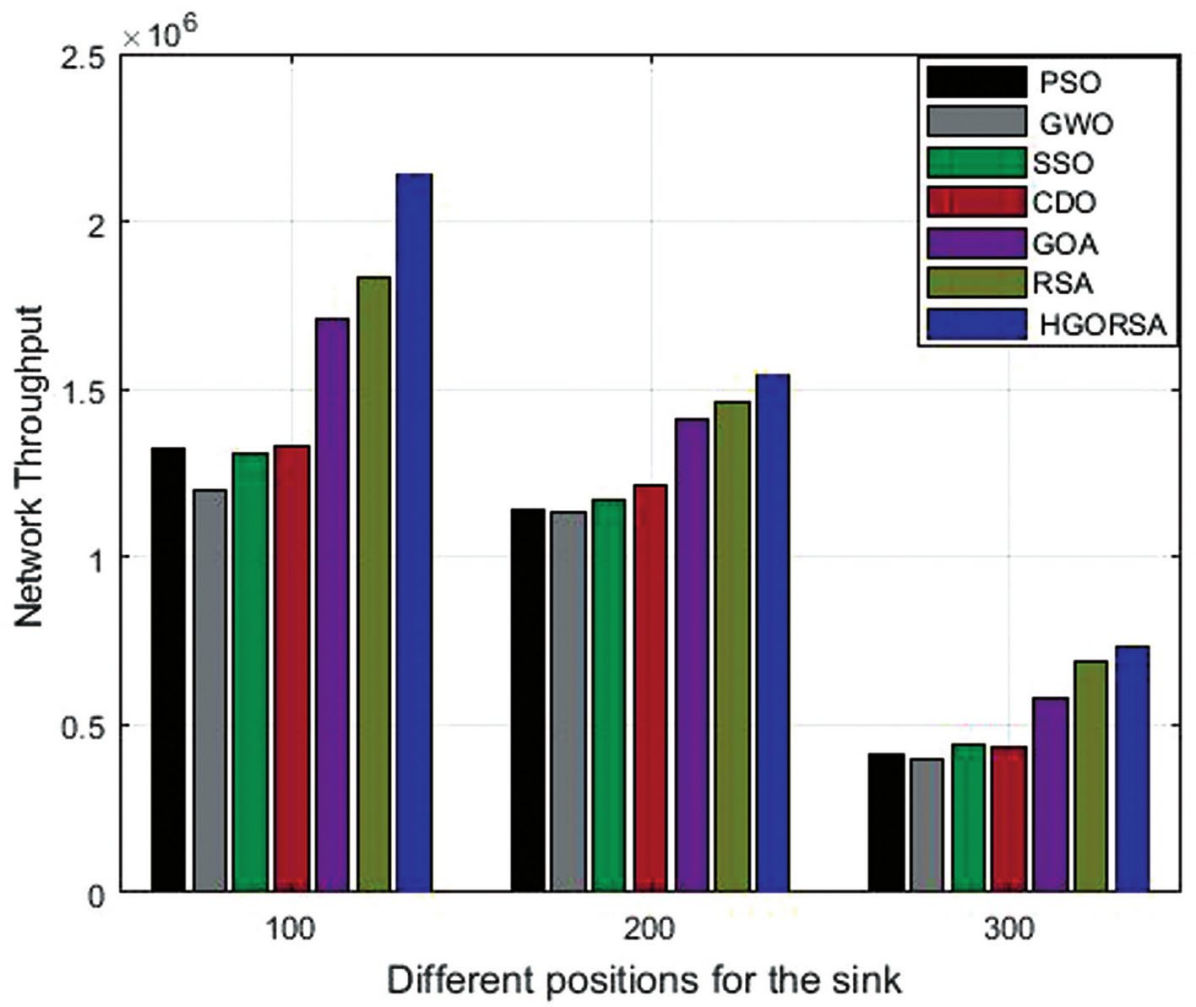
Network throughput HGORSA and other meta-heuristic algorithms when the sink is at three different positions.

The simulation outputs of HGORSA and other existing algorithms are reported in Table 10. It is clear from the values obtained that HGORSA obtains the maximum number of packets to the sink, indicating that it performs better on the network throughput of other algorithms. The simulation graphs for the throughput analysis are represented in Fig. 22.

**Table 10:** Network throughput for HGORSA and other meta-heuristic algorithms when the sink is at three different positions.

Algorithms	Field center	Field right corner	Field outside
PSO	1.42E+06	1.14E+06	4.12E+05
GWO	1.20E+06	9.18E+05	3.96E+05
SSO	1.33E+06	1.21E+06	4.31E+05
CDO	1.31E+06	1.17E+06	4.43E+05
GOA	1.71E+06	1.41E+06	5.00E+05
RSA	1.83E+06	1.46E+06	6.92E+05
HGORSA	**2.14E+06**	**1.53E+06**	**7.31E+05**

### Performance evaluation of proposed HGORSA and other meta-heuristic algorithms in terms of stability period

**Fig. 23:**
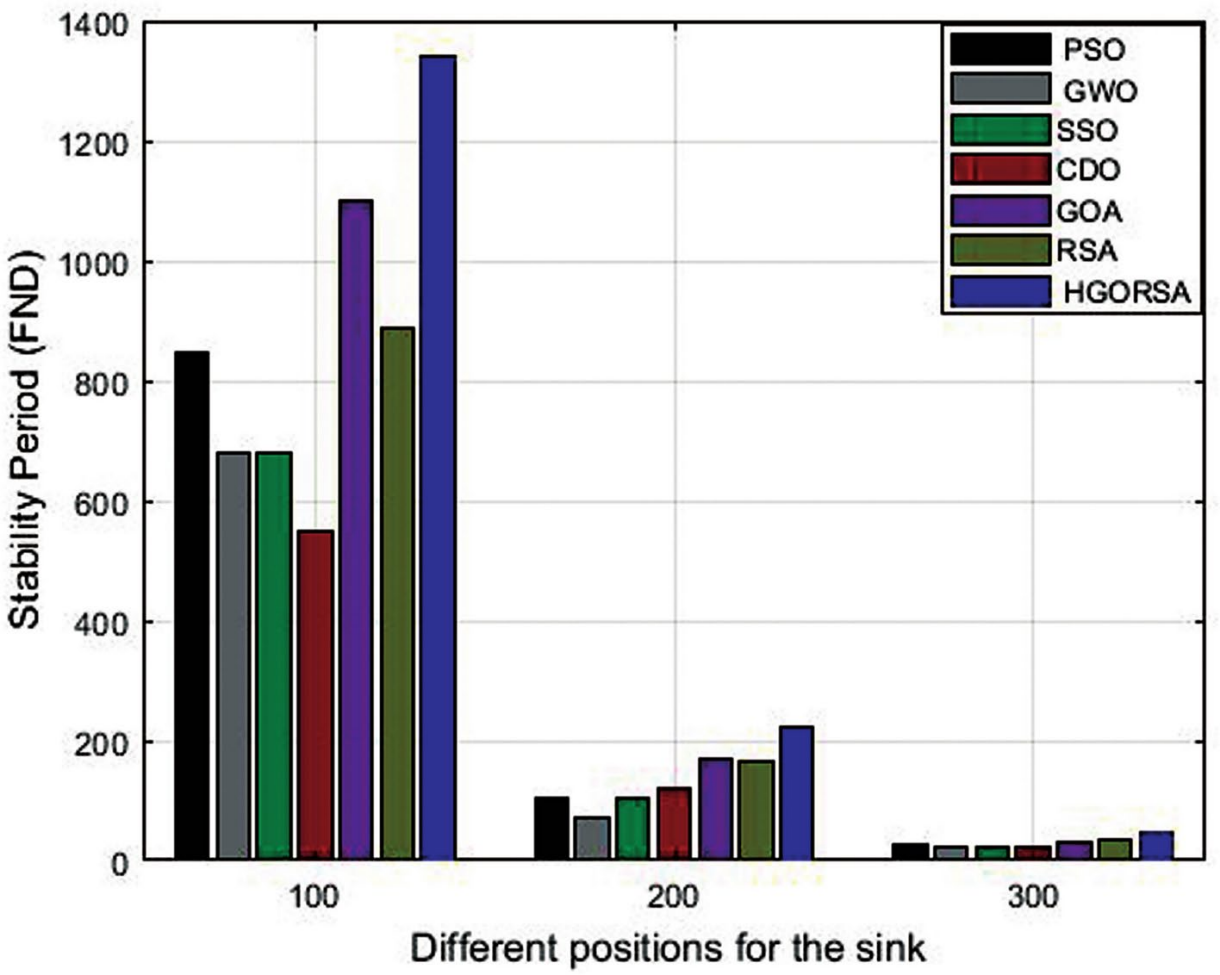
Stability period HGORSA and other meta-heuristics algorithms.

Simulation outputs from Table 11 indicate that the proposed HGORSA achieves the best results for the stability period of the network compared to other meta-heuristic algorithms. It improves the stability period by 37.3%, 49.6%, 46.8%, 55.3%, 19.1%, and 34.4%, respectively. Figure 23 plots simulation graphs of the stability period for other nature-inspired algorithms.

**Table 11:** Stability period for proposed HGORSA and other meta-heuristic algorithms when the sink is at three different positions.

Algorithms	Field center	Field corner	Field outside
PSO	848	104	25
GWO	681	72	25
SSO	681	104	22
CDO	549	122	21
GOA	1108	179	29
RSA	887	166	33
HGORSA	**1353**	**221**	**47**

### Performance evaluation of proposed HGORSA at a low and high dimension wireless sensor number

To verify the efficiency of the proposed HGORSA algorithm, an additional experiment is performed to test it on a low and high number of WSN sensors, which are specific to 50 and 500 sensors. The average results are reported after 5 runs in Table 12. The “-” sign indicates that when the proposed algorithm is applied with 50 sensors, the number of dead nodes at the three specified sink positions ranges from 4 to 11. This means that the number of dead nodes does not exceed half the total number of sensors (25). As a result, the network lifetime via the HND metric cannot be measured in this case.

**Table 12:** Performance evaluation of proposed HGORSA at 50, and 500 nodes.

Nodes	Network area	Sink Locations	Energy Consumption	HND	Dead Nodes	FND	Throughput
50	40X40	(20, 20)	52.77	-	4	1112	610000
		(40, 40)	55.29	-	7	908	468000
		(50, 50)	59.77	-	11	902	190000
500	400X400	(200, 200)	975.67	638	448	92	2.61E+06
		(400, 400)	977.35	212	449	6	2.10E+06
		(500, 500)	982.28	151	463	4	8.52E+05

The results in Table 12 show that the proposed HGORSA can obtain promising results in a low and high number of sensors. And the results reveal that HGORSA has the ability to scale with increasing network size.

### The statistical analysis of proposed HGORSA

To investigate the efficiency of the proposed algorithm, a statistical experiment was performed using the Wilcoxon test value *p* and a box plot chart.

#### Wilcoxon test *p*-value

A Wilcoxon test, also known as a Wilcoxon Signed-Rank Test, compares the means of two related groups^55^. For example, it compares the test results before and after an intervention. Since it is regarded as a nonparametric test, nonparametric data can be used with it.

The HGORSA results were compared to the best results of other algorithms used in the statistical analysis, using a non-parametric test with a significance level of 5%. The *p* values for all algorithms are shown in Tables 13, 14. Generally, it is accepted that the null hypothesis has sufficient evidence when the *p*value is less than 0.05.

**Table 13:** p-values of the HGORSA and other algorithms in terms of energy consumption.

HGORSA vs	PSO	GWO	SSO	CDO	GOA	RSA
(100,100)	0.00512	0.00512	0.00694	0.00694	0.01242	0.00512
(200,200)	0.00512	0.00512	0.00512	0.00512	0.00694	0.02852
(300,300)	0.00512	0.00512	0.00512	0.00512	0.00932	0.01242

**Table 14:** p-values of the HGORSA and other algorithms in terms of dead nodes number.

HGORSA vs	PSO	GWO	SSO	CDO	GOA	RSA
(100,100)	0.00512	0.00512	0.00512	0.00512	0.12423	0.04136
(200,200)	0.00512	0.00512	0.00512	0.00694	0.00512	0.01641
(300,300)	0.00512	0.00512	0.00512	0.00512	0.00512	0.02202

The results in Tables 13 and 14 show the values of *p* for all algorithms in terms of energy consumption and the number of dead nodes. The results indicate that there is a significant difference between the proposed algorithm and the other algorithms in most cases.

#### Box plot chart

**Fig. 24:**
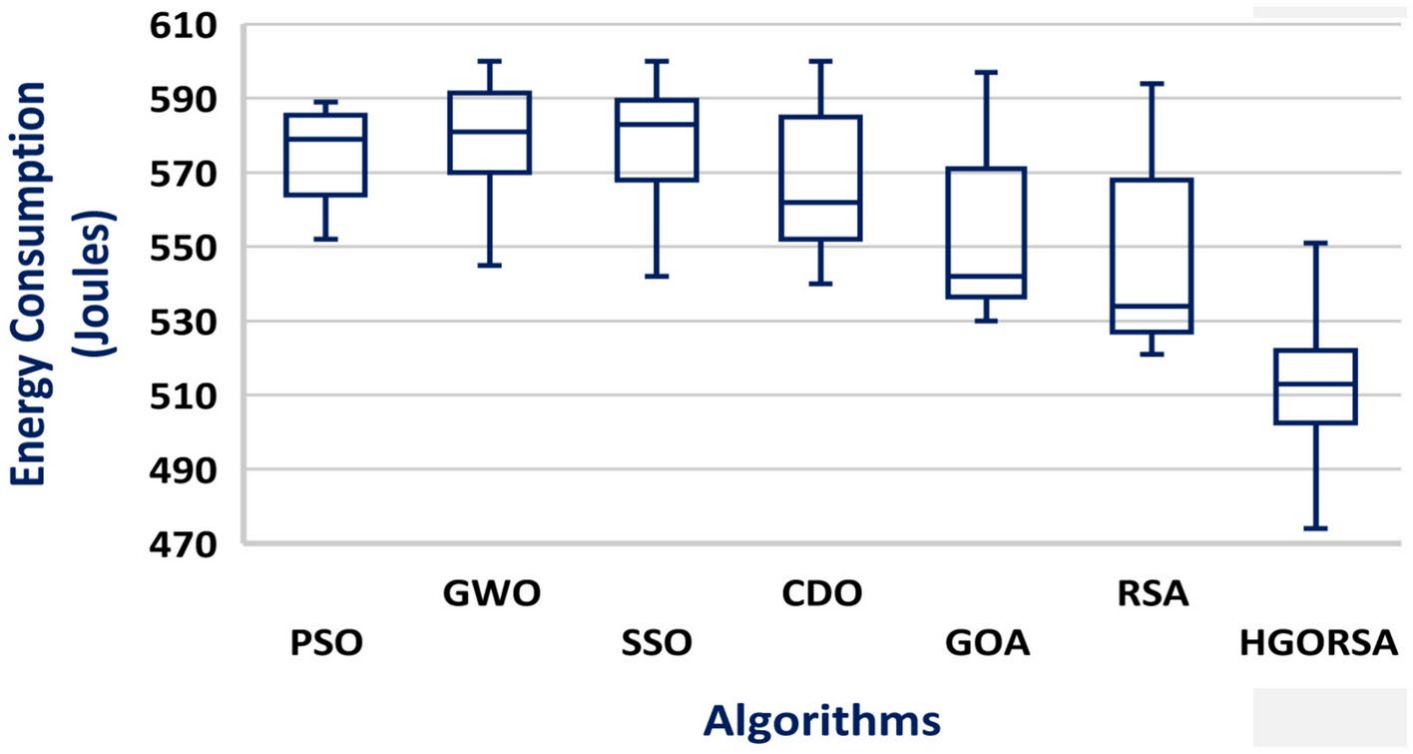
Box plot of the proposed HGORSA and other meta-heuristic algorithms, with the sink located at (100, 100), in terms of energy consumption.

**Fig. 25:**
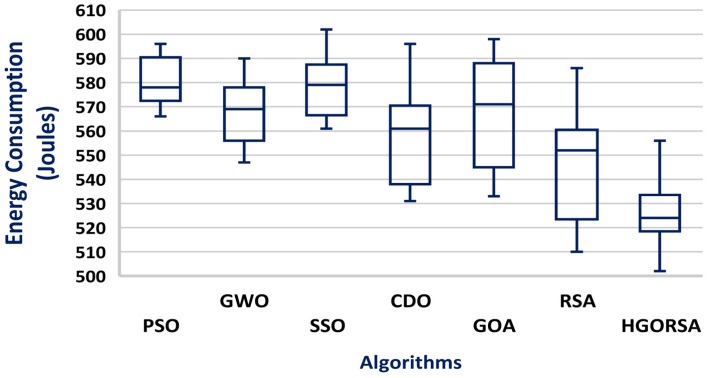
Box plot of the proposed HGORSA and other meta-heuristic algorithms, with the sink located at (200, 200), in terms of energy consumption.

**Fig. 26:**
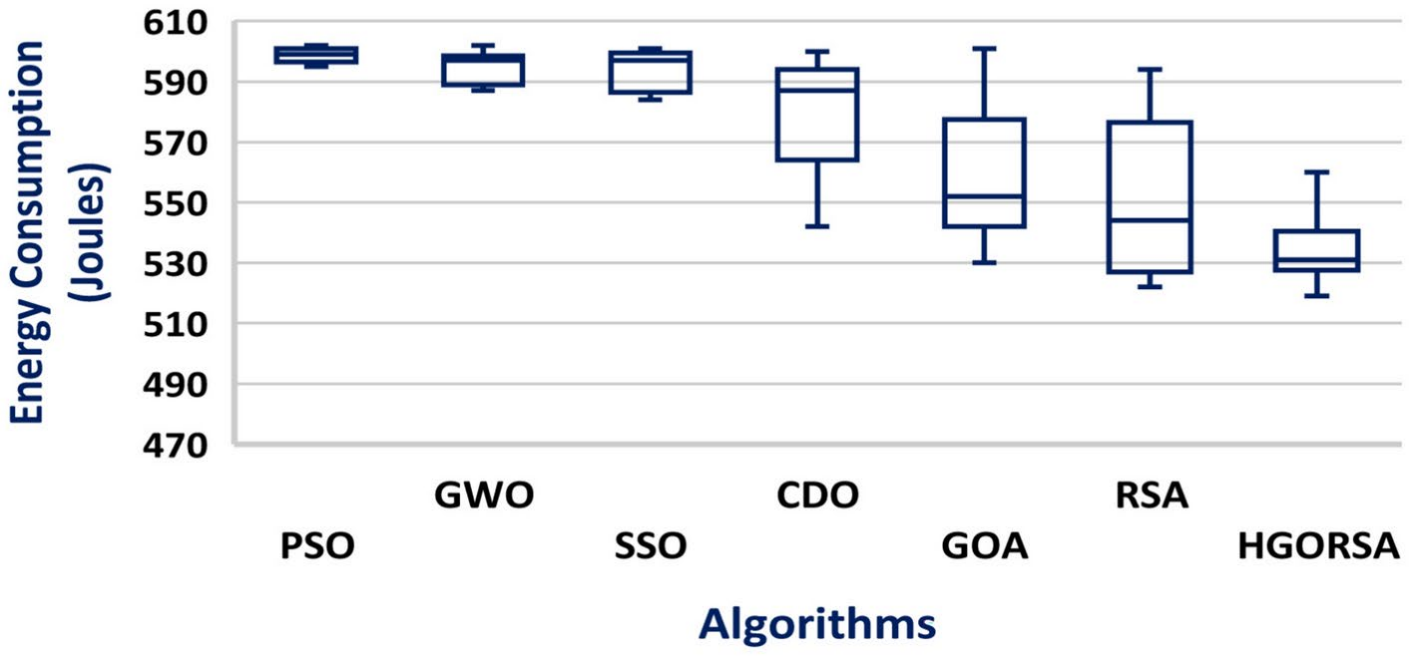
Box plot of the proposed HGORSA and other meta-heuristic algorithms, with the sink located at (300, 300), in terms of energy consumption.

**Fig. 27:**
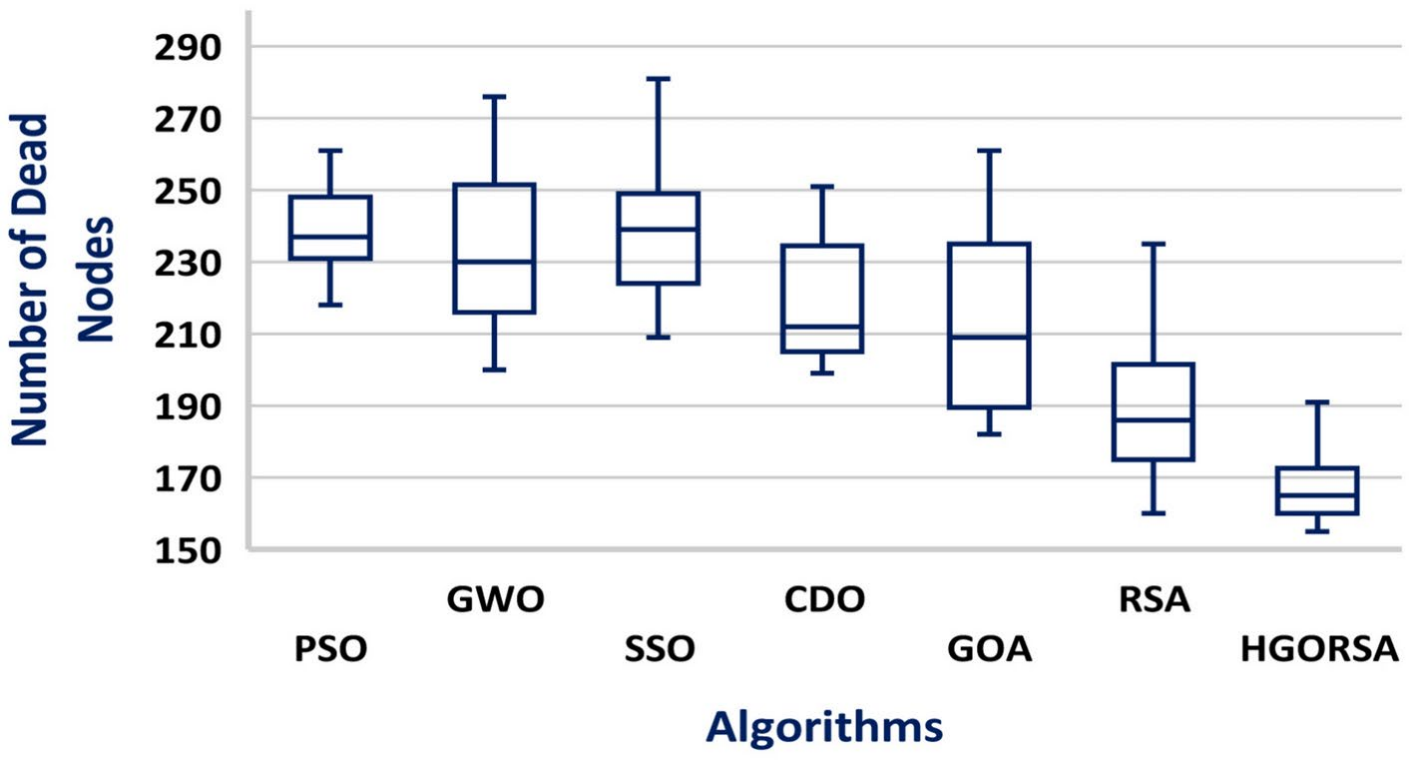
Box plot of the proposed HGORSA and other meta-heuristic algorithms, with the sink located at (100, 100), in terms of dead nods number.

**Fig. 28:**
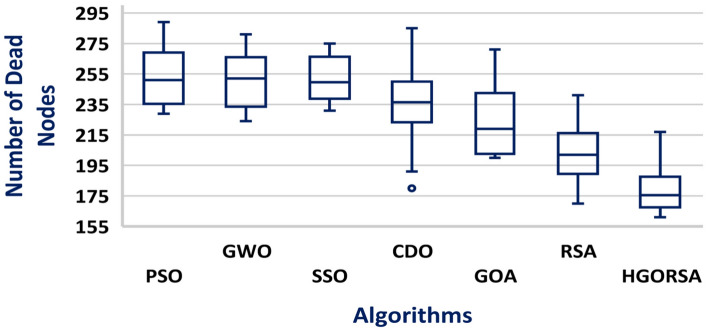
Box plot of the proposed HGORSA and other meta-heuristic algorithms, with the sink located at (200, 200), in terms of dead nods number.

**Fig. 29:**
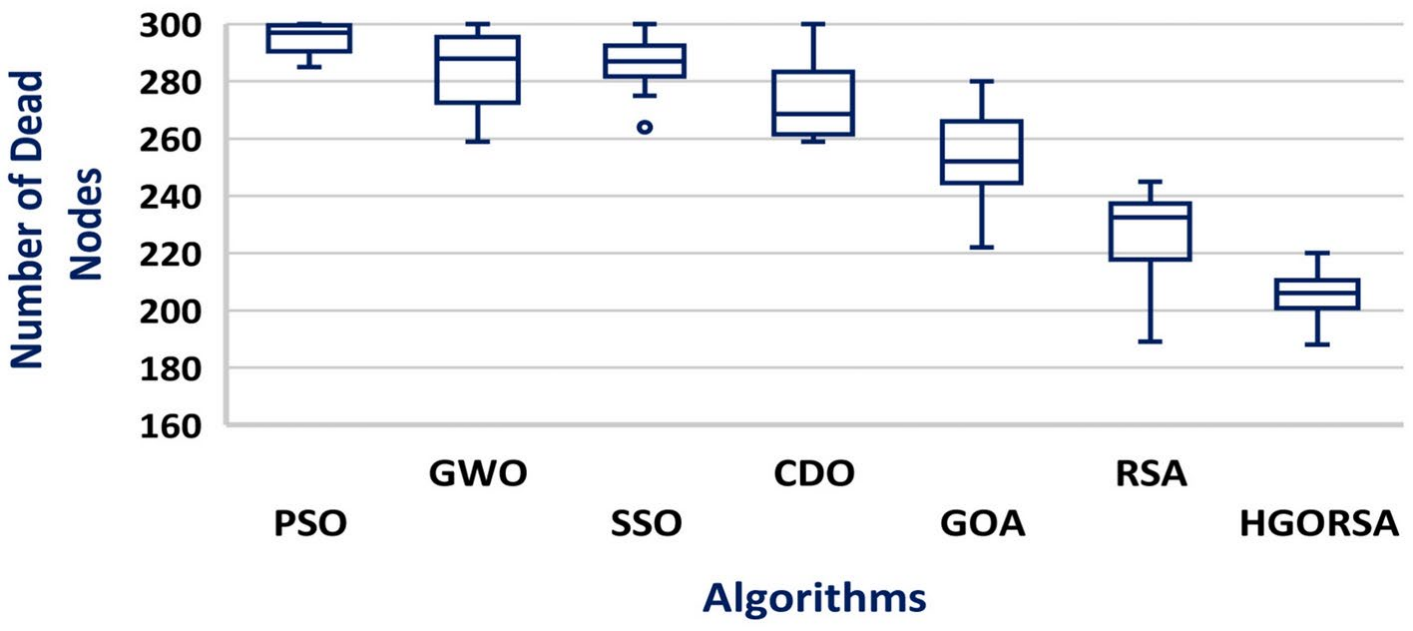
Box plot of the proposed HGORSA and other meta-heuristic algorithms, with the sink located at (300, 300), in terms of dead nods number.

A box plot is a visual representation of a dataset distribution, developed by John Tukey in 1970 and discussed in his 1977 book Exploratory Data Analysis^56^. It presents important summary statistics, including median, quartiles, and possible outliers, in a clear graphical format. Box plots provide a concise and visual way to compare various datasets, identify patterns, and summarize the distribution. The box plots of the proposed algorithm and the other meta-heuristic algorithms are presented in Figs. 24, 25, and 26, demonstrating that the median of the proposed algorithm outperforms the other algorithms in terms of energy consumption. Similarly, the box plots in Figs. 27, 28, and 29 show that the median of the proposed algorithm is superior to the other meta-heuristic algorithms in terms of the number of dead nodes.

## Conclusion and future work

This article introduces a hybrid and efficient meta-heuristic algorithm to address the challenge of clustering in WSNs, denoted HGORSA. The proposed algorithm combines the gazelle optimizer and the reptile search algorithm (RSA), resulting in improved WSN performance. Based on simulation outputs, HGORSA has demonstrated its effectiveness over PSO, GWO, SSO, CDO, GOA, and RSA. HGORSA has demonstrated percentage improvements on various performance metrics. Specifically, it achieved improvements in the stability period by 37.3%, 49.6%, 46.8%, 55.3%, 19.1%, and 34.4%, respectively. In terms of energy consumption, the improvements were 10.8%, 10.5%, 9.6%, 8.6%, 8.3%, and 3.5%, respectively. For network lifetime, the enhancements measured using the HND metric were 44.5%, 40.8%, 23.8%, 16.8%, 9.3%, and 7.2%, respectively. The reduction in the number of dead nodes was observed as 30.3%, 29.7%, 28.9%, 24.3%, 18%, and 11.5%, respectively. Furthermore, network throughput improved by 36. 4%, 43. 9%, 34. 2%, 25%, 20%, and 14. 4%, respectively. In addition, a supplementary experiment was conducted to test the efficiency of the HGORSA algorithm when the number of SNs was set to 50 and 500, using the five mentioned standard performance metrics.

Finally, the robustness of HGORSA was validated through statistical measures such as standard deviation (Std), average (Avg), worst and best values, and by providing box plots for HGORSA and the compared meta-heuristic algorithms. Based on statistical outputs, HGORSA further demonstrated its superiority over other meta-heuristics.

Although the proposed algorithm produced promising results, there is still room for improvement, such as the fact that the experiments were conducted in a simulation environment, not in real-time scenarios. Furthermore, this article does not address challenges, such as it does not consider large-scale sensor networks with more than 500 nodes. Moreover, the study has focused on homogeneous sensor network configurations, while incorporating heterogeneous sensor nodes could yield additional benefits.

For future work, the proposed algorithm will be applied to manage larger networks with more than 500 sensors and to real-time applications, allowing closer interaction with users in the physical world. Furthermore, experiments can be conducted using a large-scale real hardware architecture to handle big data.

## Data Availability

The corresponding authors will provide the data supporting the findings of the article on reasonable request.
